# A systematic review of structural neuroimaging markers of psychotherapeutic and pharmacological treatment for obsessive-compulsive disorder

**DOI:** 10.3389/fpsyt.2024.1432253

**Published:** 2025-02-13

**Authors:** Allison L. Moreau, Isabella Hansen, Ryan Bogdan

**Affiliations:** Department of Psychological and Brain Sciences, Washington University in St. Louis, Saint Louis, MO, United States

**Keywords:** structural magnetic resonance imaging, treatment, psychotherapy, pharmacotherapy, neuromarkers, obsessive-compulsive disorder

## Abstract

Identifying individual difference factors associated with treatment response and putative mechanisms of therapeutic change may improve treatment for Obsessive Compulsive Disorder (OCD). Our systematic review of structural neuroimaging markers (i.e., morphometry, structural connectivity) of psychotherapy and medication treatment response for OCD identified 26 eligible publications from 20 studies (average study total n=54 ± 41.6 [range: 11-175]; OCD group n=29 ± 19) in child, adolescent, and adult samples evaluating baseline brain structure correlates of treatment response as well as treatment-related changes in brain structure. Findings were inconsistent across studies; significant associations within the anterior cingulate cortex (3/5 regional, 2/8 whole brain studies) and orbitofrontal cortex (5/10 regional, 2/7 whole brain studies) were most common, but laterality and directionality were not always consistent. Structural neuroimaging markers of treatment response do not currently hold clinical utility. Given increasing evidence that associations between complex behavior and brain structure are characterized by small, but potentially meaningful, effects, much larger samples are likely needed. Multivariate approaches (e.g., machine learning) may also improve the clinical predictive utility of neuroimaging data.

## Introduction

Obsessive-Compulsive Disorder (OCD) has a lifetime prevalence of 1.3% [95% confidence interval: 0.9-1.8%; (1)] and brings with it tremendous individual (e.g., increased health care expenses and mortality; decreased work productivity) and societal (e.g., socioeconomic costs) burden (2–4). Effective psychological (i.e., exposure and response prevention; ERP) and medication (i.e., selective serotonin reuptake inhibitors; SSRIs) treatments are available (5–7); however, there remains tremendous variability in responsiveness to treatment [e.g., 9-76% (8)]. This variability in treatment response has inspired efforts to identify individual difference factors that may be leveraged to identify who may respond best to treatment, which may eventually contribute to more personalized medicine and greater insight into disorder and treatment heterogeneity and etiology (9). These efforts have identified some clinical predictors of treatment response, including symptom severity and hoarding pathology, while others (e.g., OCD illness duration, patient gender, age at onset, and the severity of comorbid depression or anxiety) have not been consistently associated with treatment response (10, 11). The investigation of these clinical features has had relatively little impact on treatment and has not provided clues to putative mechanisms through which therapeutic effects are generated.

There has been interest in moving beyond clinical indicators and demographic factors to identify objective and quantifiable biomarkers that are predictive of treatment response and may help inform putative mechanisms through which treatment works. While neuroimaging is not typically incorporated into clinical care for OCD, the past decade has seen an explosion of studies investigating neural predictors of treatment response as well as neural change in the context of treatment. Indeed, multiple psychiatric organizations have convened task forces to assess the existing evidence and potential for neuroimaging markers in predicting treatment response and in psychiatry more globally. The American Psychiatric Association’s working group on neuroimaging markers of psychiatric disorders concluded in their 2012 consensus report that there are promising results for predictive biomarkers of treatment response but that none yet had clinical utility (12). The World Federation of Societies for Biological Psychiatry’s task force on biological markers also argued that biomarkers can help identify treatments’ mechanisms of action (13). Recent work has continued to investigate the possibility of diagnostic and prognostic neuroimaging biomarkers for psychiatric disorders (e.g., (14–16); see (17) for an umbrella review of OCD diagnostic biomarkers). However, the literature is dispersed with a variety of approaches used and there is a need for systematic reviews to synthesize this work. While comprehensive reviews have been published recently for other disorders, including depression (18), an up-to-date systematic review of psychotherapy and medication treatments for OCD is needed.

### Neuroimaging of OCD and treatment response

#### Neuroimaging of OCD

Initial theoretical models of OCD neurobiology emphasized that alterations in a cortico-striato-thalamo-cortical (CSTC) circuit may contribute to the expression of OCD or arise following its expression (19–21). This circuit is involved in motor and response inhibition, affective and reward processing, and working memory and executive function, all of which are impaired in OCD. More recently the CSTC model has been updated to include additional regions, namely the amygdala (20), for its role in fear extinction, and the parietal cortex, for its role in cognitive control(see (22, 23) for recent reviews). Large scale international consortiums, such as the Enhancing Neuroimaging Genetics through Meta-Analysis (ENIGMA) consortium (24), have integrated smaller patient studies to more reliably estimate brain structure correlates of OCD (25) and other psychopathology. In relatively large samples (OCD patients ns: 874-2,278; control ns: 789-2,093), the ENIGMA OCD working group found that adult OCD is associated with: 1) smaller hippocampal and larger pallidal volume (26); 2) thinner bilateral inferior parietal cortex and smaller left transverse temporal surface area (27); 3) lower fractional anisotropy (FA) and higher radial diffusivity (RD) in the sagittal stratum and posterior thalamic radiation as well as higher mean diffusivity (MD) in the sagittal stratum (28). Pediatric OCD patients (OCD patients ns: 174-407; control ns: 144-324), like adult patients, had thinner left inferior parietal cortex, as well as thinner bilateral superior parietal, and left lateral occipital cortices, which were not seen in adult patients, but no significant differences in surface area (27), subcortical volume (26), or white matter microstructure (28). Notably, medication and/or severity may contribute to these adult and pediatric findings as a large number of included patients were medicated (43-50% of patients in studies analyzing medication groups) and no significant differences in volume, cortical thickness, or surface area were observed when restricting analyses to unmedicated adult patients and only one finding was observed for unmedicated pediatric patients (larger thalamic volume) (26, 27). Furthermore, multivariate machine learning analyses using cortical thickness, surface area, and subcortical volume to classify patients versus controls only performed above chance when grouping patients according to medication status (29). These findings highlight the potential effects of medications on brain structure and the importance of controlling for medication status. ENIGMA’s cross-sectional findings in medicated patients need to be combined with the results of studies explicitly designed to probe treatment, both medications and psychotherapy, which crucially include pre-treatment assessments.

#### Neuroimaging of treatment response

Neuroimaging studies of treatment outcome typically utilize pre-treatment scans to predict treatment outcome or evaluate neural changes between pre- and post-treatment scans. These studies have used a variety of imaging-derived neural phenotypes, including functional and structural MRI. While fMRI studies have been the most common and have already been extensively reviewed in the context of OCD treatment (e.g. (30–32)), emerging evidence suggests that their low reliability may not be suitable for individual differences research [e.g., resting state: (33), task-based: (34)]. Structural MRI, however, has not suffered from this issue, with higher test-retest reliability than fMRI (34). Structural MRI also has other advantages over fMRI that make it valuable for studying treatment response. The expense of MRIs represents a hurdle for clinical feasibility, particularly in the context of expected small effects. However, many individuals undergo a structural MRI of the brain for other medical reasons that become part of their medical record. These scans may be leveraged for psychiatric purposes, further increasing the value of existing MRIs. Functional MRIs, on the other hand, are not routinely acquired for medical care. They also require more equipment, expertise, and processing efforts; therefore, structural scans may be more practical for clinical questions. It is also possible that in the near future structural analyses will be automatically constructed on MRI scanners (e.g., CorticoMetrics’ THINQ, which is an FDA-approved version of the FreeSurfer neuroimaging analysis software package for clinical use), increasing the accessibility of structural brain metrics.

While the mechanism of change for fMRI studies is intuitive [i.e., treatment affects neural activation through altering synaptic levels of neurotransmitters (for medications) or other molecular mechanisms of learning (for psychotherapy)], the mechanism of change for structural MRI studies is less clear. For medications, SSRIs, the primary class of medications for OCD, have been shown to change synaptic transmission and post-synaptic transcription growth factors and stimulate neurogenesis (35–38). For psychotherapy, treatment involves learning new cognitive and behavioral strategies, and learning processes, in general, have been associated with changes in brain structure (39); therefore, it is possible that psychotherapy could lead to structural changes in the brain. In fact, the most effective psychotherapy for OCD, exposure and response prevention, is a form of extinction learning, and studies of healthy controls have shown that structural differences (namely in the ventromedial prefrontal cortex) may be associated with extinction recall abilities (40). Thus, it would be reasonable for improvements in extinction recall following exposure and response prevention to be correlated with structural changes in these brain regions.

### Current review

To date, there has not yet been a comprehensive review for structural markers of OCD treatment response. Existing reviews that include such studies (30–32, 41–44) only contain a handful of such studies, at most. Therefore, the aim of this paper is to provide a systematic review of the literature on structural neuroimaging markers of treatment response for OCD, including pre-treatment response predictors as well as changes after treatment.

## Methods

A systematic review on brain structure correlates of OCD treatment was conducted according to the 2020 Preferred Reporting Items for Systematic Reviews and Meta-Analyses (PRISMA) guidelines (45) (see **Supplementary Table 1** for PRISMA checklist). Database searches were conducted by author AM on September 19, 2020 and subsequently revisited prior to manuscript submission on January 28, 2022, May 21, 2022, and July 23, 2022. The study protocol was not preregistered; see **Supplementary Data Sheet 1** for protocol details.

### Study selection

#### Search strategy and eligibility criteria

A systematic search using PsycINFO, PubMed, and Web of Science and the reference lists of included articles was conducted to identify studies in which neuroimaging was used to predict OCD treatment response or neuroimaging change was measured during the course of OCD treatment. Inclusion criteria included: peer-reviewed empirical journal articles, published in English, examines OCD treatment, and measures brain structure. Dates of publication and age of subjects were not restricted. Exclusion criteria included case studies and studies that acquired brain scans of patients already undergoing treatment or that included treatment status as a covariate for other research questions.

#### Search

The following search terms were used: (“obsessive compulsive disorder” OR “obsessive-compulsive disorder”) AND (“treatment” OR “treatment response” OR “treatment outcome” OR “therapy” OR “psychotherapy” OR “counseling” OR “intervention” OR “empirically supported treatment” OR “empirically based treatment” OR “medication” OR “psychopharmacology” OR “pharmacotherapy” OR “cognitive behavioral therapy” OR “exposure and response prevention” OR “exposure therapy” OR “SSRIs” OR “neurostimulation” OR “deep brain stimulation” OR “DBS” OR “transcranial magnetic stimulation” OR “TMS” OR “electroconvulsive shock therapy” OR “ECT” OR “vagus nerve stimulation” OR “psychosurgery” OR “capsulotomy” OR “cingulotomy”) AND (“neuroimaging” OR “magnetic resonance imaging” OR “MRI” OR “structural magnetic resonance imaging” OR “structural MRI” OR “volume” OR “surface area” OR “cortical thickness” OR “diffusion” OR “diffusion weighted imaging” OR “diffusion tensor imaging”). Keywords were searched for in “MeSH subject headings” (PsycINFO), “Title/Abstract” (PubMed), and “Topic” (Web of Science).

#### Study selection

Search results from all three databases were exported to Mendeley (Version 1.19.8, Mendeley Ltd.) and then abstrackr [abstrackr.cebm.brown.edu; (46)]. During the first round of screening, titles, abstracts, and keywords were reviewed by AM and IH to assess study eligibility (see Abstract screening tool in **Supplementary Data Sheet 2**). The first 100 search results were screened by both AM and IH to determine interrater reliability. The agreement rate was 95 percent, with four of the remaining five articles marked “no” by one rater and “requires full-text review” by the other; all discordant articles were ultimately excluded after full-text review. After establishing sufficient reliability and resolving any inconsistencies in screening approach, the remaining results were screened by either AM or IH. For articles deemed of potential relevance, the full text was reviewed by AM and RB was consulted as needed.

### Data collection process

Study characteristics, methods, and results were extracted from included studies by AM, who has specialized clinical training in OCD and expertise in structural MRI. Neuroimaging or statistical files did not need to be obtained from study authors because the heterogeneity of methods (e.g., ROI vs whole brain) and approaches precluded a meta-analysis. All studies measured OCD symptom severity using the clinician-administered Yale-Brown Obsessive-Compulsive Scale (Y-BOCS) (47), or the Children’s Yale-Brown Obsessive-Compulsive Scale (CY-BOCS) (48) for pediatric patients, which each generate total scores, ranging from 0-40, as well as obsession and compulsion subscales ranging from 0-20 each. Outcome variables were Y-BOCS scores or morphometry measurements, depending on study design.

## Results

**Figure 1:**
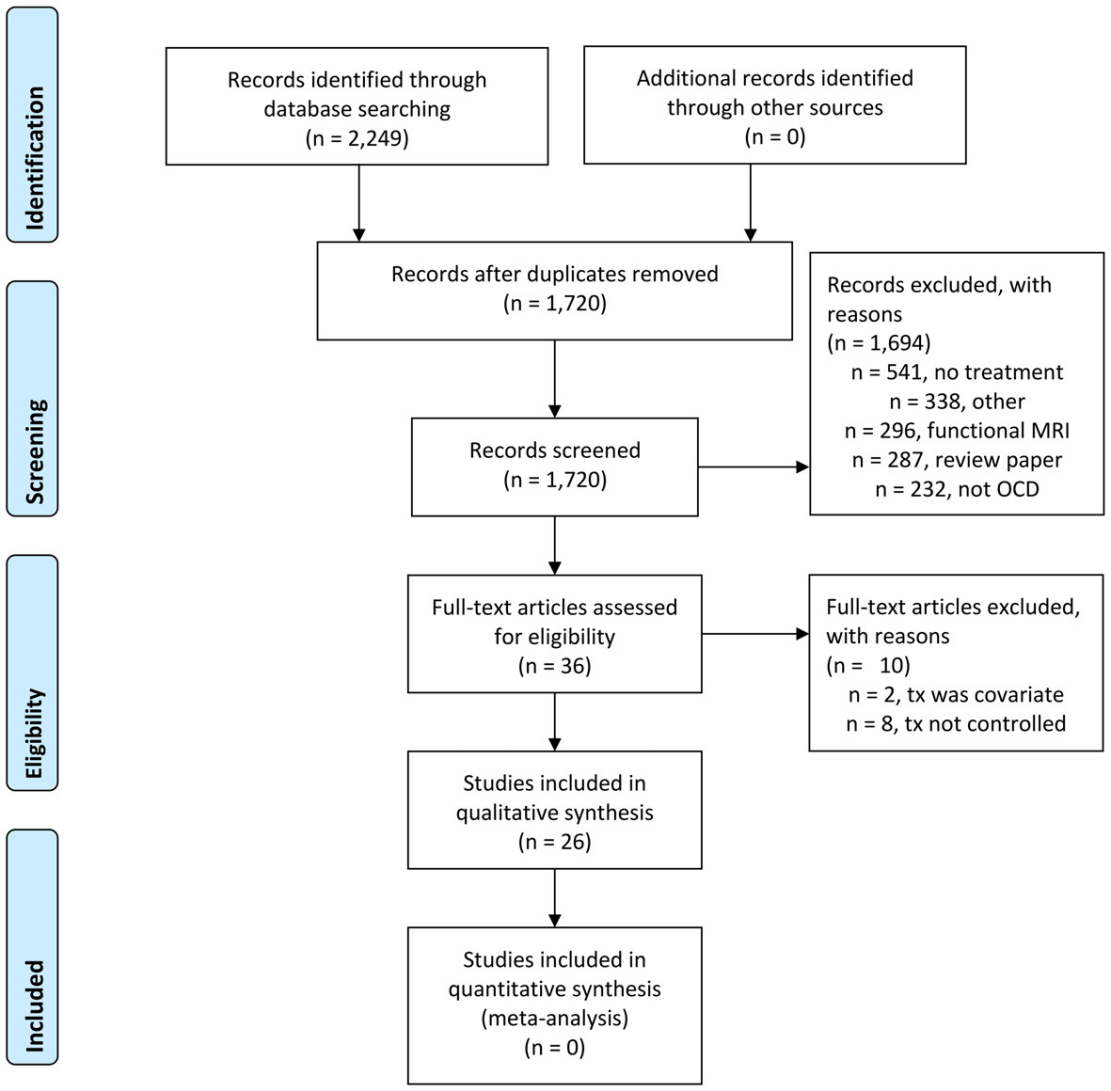
PRISMA flow diagram of systematic search process.

The systematic search identified 1,720 unique peer-reviewed journal articles, of which 26 publications met full inclusion criteria (**Figure 1**; **Table 1**). Most identified publications (i.e., 21 of 26) were published after 2012. Of these 26 articles, 6 analyzed data from a sample that had already been published on (e.g., using different techniques, reporting on different analyses, or conducting longitudinal analyses). Thus, there were 20 independent samples; total sample sizes ranged from 11 to 175 participants (mean ± SD = 54 ± 41.6), with OCD group sizes ranging from 11to 85 before treatment (mean ± SD = 29 ± 18.6) and 10 to 74 after treatment (mean ± SD = 26 ± 16). Most publications included healthy control groups (n=21). Thirty-one percent of publications (i.e., 8/26) were conducted in child/adolescent samples.

**Table 1:** Study characteristics.

Study	Sample size	Ages	Neuroimaging details	Longitudinal design?	Comorbidities allowed?	Treatment protocol	Y-BOCS
PSYCHOTHERAPY STUDIES							
Adult studies							
Brecke et al. (49)	Total = 62 *(48 w/post-tx scan)* 32 OCD *(26 w/post-tx scan)* 30 Controls *(22 w/post-tx scan)*	*Mean ± SD:* OCD: 30.3 *±* 9.0 Controls: 31.0 *±* 10.5	Diffusion 3T GE Discovery MR750	Yes	Yes	4-day concentrated ERP (Bergen 4-Day Treatment)	*Before tx:* 27.1 ± 3.9 *After tx:* 11.4 ± 6.4 *3-mo follow-up:* 10.6 ± 6.4
Cao et al. (50)	Total = 84 34 OCD 50 Controls	*Range:* 18-50 *Mean ± SD:* OCD: 27.6 ± 6.7 Controls: 28.2 ± 6.8	Diffusion 3T Siemens Trio	Yes	No	Fourteen 60-minute CBT (w/ERP) sessions over 12 weeks	*Before tx:* Responders: 23.6 ± 6.5 Non-responders: 25.8 ± 4.7 *After tx:* Responders: 8.7 ± 3.8 Non-responders: 20.5 ± 6.8
Zhong et al. (51)	Total = 175 85 OCD *(56 completed CBT)* 90 Controls	*Range*: 18-50	Diffusion 3T Siemens MRI Scanner	Yes	No	Fourteen 60-minute CBT (w/ERP) sessions over 12 weeks	*Before tx:* 23.6 ± 5.7 *After tx:* 11.4 ± 5.8
Atmaca et al. (52, 53)^†^	Total = 24 12 OCD 12 Controls	*Mean ± SD*: OCD: 30.1 ± 4.9 Controls: 29.5 ± 4.7	Volume 1.5T GE Signa	Yes	Yes – only depression	16 weekly sessions of ERP	*Before tx:* 22.7 ± 4.3 *After tx:* 14.4 ± 5.3
Tsuchiyagaito et al. (54)	Total = 37 *(31 w/MRI)* 15 OCD+ASD *(13 w/MRI)* 22 OCD only *(18 w/MRI)*	*Range*: 17-50 *Mean ± SD:* OCD only: 34.1 ± 7.4	Volume 3T GE Discovery MR750	No	Yes	11-20 weekly 50-minute CBT sessions	*Before tx* (OCD only patients): 25.9 ± 4.0
Hashimoto et al. (55) *all subjects also on SSRIs	Total = 69 39 OCD 30 Controls	*Mean ± SD*: Responders: 35.7 ± 7.2 Non-responders: 32.5 ± 7.7 Controls: 32.5 ± 6.7	Volume 1.5T Philips Gyoro Scan Interu	No	No	12 weekly sessions of ERP	*Before tx:* Responders: 33.3 ± 4.2 Non-responders: 33.0 ± 5.3 *After tx:* Responders: 16.0 ± 3.3 Non-responders: 30.0 ± 5.5
Fullana et al. (56) *all subjects also on medications	Total =160 74 OCD 86 Controls	*Range*: 18-61 *Mean ± SD:* OCD: 34.1 ± 9.2 Controls: 33.6 ± 9.8	Volume, cortical thickness 1.5T GE Signa	No	Yes	20 weekly sessions of ERP	*Before tx:* 22.2 ± 5.1 *After tx:* 15.4 ± 6.7
Pediatric studies							CY-BOCS
Pagliaccio et al. (57)	Total = 55 28 OCD 27 Controls	*Range:* 7-18 *Mean ± SD:* OCD: 11.3 ± 3.2 Controls: 12.1 ± 3.3	Volume, cortical thickness, diffusion 3T GE Signa	Yes	Yes – anxiety disorders only	12-16 60-minute sessions of CBT (w/ERP)	*Before tx:* 24.3 ± 5.1 *After tx:* 15.7 ± 7.7
Huyser et al. (58)	Total = 37 17 OCD 20 Controls	*Range:* 8-19 *Mean ± SD:* OCD: 13.8 *±* 2.8 Controls: 14.6 *±* 2.6	Volume 3T Philips Intera	Yes – end of treatment and 1.5 years later *follow-up to Huyser et al. (59)	Yes, except psychosis or bipolar disorder	16 weekly sessions of CBT (w/ERP)	*Before tx:* 25.4 ± 5.3 *After tx:* 12.8 ± 9.0 *Follow-up:* 10.3 ± 8.9
Huyser et al. (59)	Total = 58 29 OCD *(26 w/post-tx scan)* 29 Controls *(27 w/post-tx scan)*	*Range:* 8-19 *Mean ± SD:* OCD: 13.8 *±* 2.6 Controls: 13.6 *±* 2.7	Volume 3T Philips Intera	Yes – end of treatment	Yes, except psychosis or bipolar disorder	16 weekly sessions of CBT (w/ERP)	*Before tx:* 24.9 ± 5.0 *After tx:* 13.0 ± 9.2
Rosenberg et al. (60)	Total = 11 All OCD	*Range:* 8-17 *Mean ± SD:* 12.9 *±* 3.2	Volume 1.5T GE Horizon	Yes	Yes – anxiety disorders, ADD, dysthymia, trichotillomania	14 60-min ERP sessions over 12 weeks	*Before tx:* 22.5 ± 4.2 *After tx:* 10.5 ± 5.5
MEDICATION STUDIES							
Adult studies							
Atmaca et al. (61, 62)^†^	Total = 28 14 OCD 14 Controls	*Mean ± SD:* OCD: 33.0 ± 3.8 Controls: 30.4 ± 4.1	Volume 1.5T GE Signa	Yes – for OCD group, Controls only had baseline scan	Yes – depression	SSRIs or clomipramine, 12 weeks	*Before tx:* 26.4 ± 4.2 *After tx:* 10.1 ± 4.7
Tang et al. (63)	Total = 34 18 OCD 16 Controls	*Range:* 18-60 *Mean ± SD:* OCD: 27.3 ± 10.4 Controls: 26.8 ± 9.8	Volume 3T	Yes – OCD treatment responders only (n=11) scanned twice	No	Sertraline, 12 weeks	*Before tx:* 29.7 ± 3.4 *After tx:* Not reported
Yun et al. (64)	Total = 131 56 OCD *(25 responders,* *31 non-responders)* 75 Controls	*Mean ± SD:* Responders: 23.5 ± 5.4 Non-responders: 27.2 ± 5.9 Controls: 25.1 ± 5.5	Surface area, cortical thickness 3T Siemens Magnetom	No	Unclear	SSRIs (escitalopram or fluoxetine), serotonin-dopamine antagonist if needed, 4 months	*Before tx:* Responders: 27.2 ± 5.0 Non-responders: 28.2 ± 5.6 *After tx:* Responders: 12.8 ± 4.2 Non-responders: 24.4 ± 5.7
Narayanaswamy et al. (65)	Total = 29 All OCD	*Mean ± SD:* 26.9 ± 7.0	Volume 1.5T Siemens Magnetom	No	Yes	SSRIs, 2-5 years	*Before tx:* 27.3 ± 8.3 *After tx:* 12.9 ± 9.8
Fan et al. (66)	Total = 50 27 OCD *(only 15 received tx)* 23 Controls	*Range:* 18-54 *Mean ± SD:* OCD: 25.5 ± 7.0 Controls: 28.8 ± 7.6	Diffusion 1.5T GE MRI scanner	Yes	No	SSRIs, 12 weeks	*Before tx:* 22.0 ± 4.9 *After tx:* 9.9 ± 3.2
Yoo et al. (67)	Total = 26 13 OCD 13 Controls	*Mean ± SD:* OCD: 27.8 ± 7.3 Controls: 26.9 ± 7.0	Diffusion 1.5T Philips MRI Scanner	Yes	Yes	Citalopram, 12 weeks	*Before tx:* 30.2 ± 4.6 *After tx:* 16.6 ± 6.4
Pediatric studies							CY-BOCS
Szeszko et al. (68) +	Total = 22 11 OCD 11 Controls	*Range*: 6-12.5 *Mean ± SD:* OCD: 11.8 ± 3.0 Controls: 13.3 ± 2.4	Volume 1.5T GE Horizon	Yes	Yes – although most excluded	Paroxetine, 16 weeks	*Before tx:* 28.5 ± 6.4 *After tx:* 18.2 ± 7.2
Gilbert et al. (69)+	Total = 42 21 OCD *(10 received tx)* 21 Controls	*Range:* 8-17 *Mean ± SD:* OCD: 12.4 ± 2.9 Controls: 12.5 ± 2.6	Volume 1.5T GE Horizon	Yes – for 10 from OCD group. other 11 did not receive paroxetine.	Yes	Paroxetine, 16 weeks	*Before tx:* 31.3 ± 4.4 *After tx:* 20.6 ± 5.9
PSYCHOTHERAPY AND MEDICATION STUDIES							
Adult studies							
Hoexter et al. (70)	Total = 41 29 OCD in test sample *(14 Fluoxetine, 15 CBT)* 12 OCD in validation sample	*Range:* 18-65 *Mean ± SD:* Test sample: 33.2 ± 10.6 Validation sample: 33.5 ± 11.4	Cortical thickness 1.5T GE Signa	No – only used baseline scans from RCT to predict tx response	Yes – except psychosis, suicide risk or substance abuse	Randomly assigned to 12 weeks of fluoxetine or group CBT (2-hr ERP sessions)	*Before tx (test sample):* Responders: 26.1 ± 3.5 Non-responders: 25 ± 6.5 *After tx (test sample):* Responders: 10.6 ± 3.4 Non-responders: 21.2 ± 7.8
Hoexter et al. (71)	Total = 29 14 Fluoxetine 15 Group CBT 0 Controls	*Range:* 18-65 *Mean ± SD:* 33.2 ± 10.6	Volume 1.5T GE Signa	No – only used baseline scans from RCT to predict tx response	Yes – except psychosis, suicide risk or substance abuse	Randomly assigned to 12 weeks of fluoxetine or group CBT (2-hr ERP sessions)	*Before tx:* Fluoxetine: 23.5 ± 4.9 CBT: 27.3 ± 5.2 *After tx:* Fluoxetine: 14.4 ± 6.3 CBT: 18.4 ± 9.4
Hoexter et al. (72)	Total = 74 38 OCD *19 each for fluoxetine and group CBT (only 13 each w/post-tx MRI)* 36 Controls	*Range:* 18-65 *Mean ± SD:* OCD: 31.5 ± 10.2 Controls: 27.8 ± 7.8	Volume 1.5T GE Signa	Yes – at end of treatment	Yes – except psychosis, suicide risk or substance abuse	Randomly assigned to 12 weeks of fluoxetine or group CBT (2-hr ERP sessions)	*Before tx:* 25.1 ± 5.2 *After tx:* 16.3 ± 8.1
Pediatric studies							
Vattimo et al. (73)	Total = 57 29 OCD *12 Fluoxetine* *17 Group CBT* 28 Controls	*Range*: 7-17 *Mean ± SD:* Responders: 12.1 ± 2.6 Non-responders: 12.3 ± 2.4 Controls: 11.5 ± 2.3	Volume 3T Philips Achieva	No	Yes – except suicidal ideation, schizophrenia or bipolar disorder	Randomly assigned to 14 weeks of fluoxetine *or* group CBT	*Before tx:* Responders: 25.6 ± 4.4 Non-responders: 28.5 ± 5.4 *After tx:* Responders: 8 ± 5.9 Non-responders: 25.6 ± 4.8
Lázaro et al. (74)*	Total = 30 15 OCD 15 Controls	*Range:* 9-17 *Mean ± SD*: OCD: 13.7 ± 2.5 Controls: 14.3 ± 2.5	Volume 1.5T GE Signa	Yes	No	6 months of SSRIs *and* CBT (w/ERP)	*Before tx:* 25.9 ± 5.6 *After tx:* 9.7 ± 8.0

tx, treatment; SD, standard deviation; CBT, cognitive behavioral therapy; ERP, exposure and response prevention; ADD, attention deficit disorder; RCT, randomized clinical trial; SSRI, selective serotonin reuptake inhibitor. Y-BOCS and C-YBOCS scores listed are for OCD patients only. ^†^The two Publications (52, 53) are from the same study and sample. Publications (61, 62) are also from the same study and sample. It is unclear if there is sample overlap between (51/52) and (60/61). *All subjects in (74) were given both fluoxetine and exposure and response prevention, so the effects of treatment type cannot be determined. + These samples overlap. Most of the Szeszko et al. (68) sample was part of the Gilbert et al. (69) sample.

Study treatments were relatively equally divided between psychotherapy (n=17) and medication (n=14) publications; five of these evaluated both psychotherapy and medication. Results are described below in subsections for each treatment type. The original search included any form of treatment (e.g., psychotherapy, medication, psychosurgery, or brain stimulation). However, given the small sample size of surgical and brain stimulation treatment studies, their smaller number, varying procedures (e.g., cingulotomy, capsulotomy, deep brain stimulation, transcranial magnetic stimulation), and the typical assessment of direct consequences of the operation or brain stimulation (e.g., post-surgery lesion measurement), these studies were not included in the systematic review.

Sixteen of the 26 publications reported on data acquired on MRI scanners with 1.5 Tesla field strength, with the remainder coming from 3T scanners. Twelve of the total 26 publications estimated associations between brain structure measured at pre-treatment and clinical response. In studies that included pre- and post-treatment scans of patients (n=17), six rescanned controls at the equivalent of post-treatment. Seventeen studies reported on brain structure differences from pre- to post-treatment and their associations with clinical outcomes.

The majority of publications evaluated gray (n=18) or white (n=4) matter volumes. Other brain structure metrics examined included white matter structural integrity (n=6), cortical thickness (n=4), and surface area (n=1). Studies examined associations across the entire brain (n=8), specific regions of interest (ROIs) (n=11), or adopted both approaches (n=7). The following specific ROIs were examined across studies (n= number of publications): orbitofrontal cortex (n=10), anterior cingulate cortex (n=5) and cingulum white matter tract (n=2), thalamus (n=7), caudate (n=5), amygdala (n=3), putamen (n=3), hippocampus (n=2), pallidum (n=2), parahippocampal gyrus (n=2), pituitary gland (n=2), dorsolateral prefrontal cortex (n=1), precentral gyrus (n=1), superior frontal and middle frontal gyri (n=1), parietal cortex (n=1), supramarginal gyrus (n=1), subcallosal cortex (n=1), sagittal stratum and posterior thalamic radiation white matter tracts (n=1), corpus callosum (n=1), and external and internal capsule white matter (n=2).

### Therapy studies

#### Study design summary

##### Studies and samples

The 17 psychotherapy OCD treatment outcome publications (n=6 from pediatric samples) originated from 13 independent samples (n=5 from pediatric samples; **Table 1**). Total sample sizes ranged from 11 to 175 participants (mean ± SD = 60 ± 45), with OCD group sizes ranging from 11 to 85 before treatment (mean ± SD = 32 ± 20) and 11 to 74 after treatment (mean ± SD = 29 ± 16).

##### Treatments

Samples contained patients who were: treatment naïve (n_samples_=2; n_publications_=4); previously treated with medication but not currently using medication or never having used medication to treat OCD (n_samples_=5; n_publications_=6); a mixture of those currently taking medication and those who were not (n_samples_=4; n_publications_=4); all concurrently taking medications during therapy (n_samples_=3; n_publications_=3). Of the treatment outcome studies allowing concurrent medication, two were conducted among medication non-responders, who remained on their medication (55, 56); one began four patients on SSRIs mid-therapy following lack of response to psychotherapy alone (57); one provided concurrent combination treatment (74); a longitudinal study began four patients on medications after psychotherapy ended (58); and the remaining two studies permitted patients already on medications to maintain stable doses throughout therapy (49, 54).

##### Brain phenotypes and analytic strategy

Six studies (n_samples_=6) estimated associations across the whole brain using whole-brain voxel-based morphometry (n_studies_=3) or voxel-based diffusion analyses (n_studies_=1) with others analyzing all atlas ROIs (n_studies_=2). Six publications (n_samples_=5) analyzed specific ROIs only. Five publications (n_samples_=3) used both ROI and whole brain voxel-wise analyses. The following specific ROIs were examined across studies (n=number of publications): orbitofrontal cortex (n=8), anterior cingulate cortex (n=4) and cingulum white matter tract (n=2), thalamus (n=5), caudate (n=4), amygdala (n=2), putamen (n=3), hippocampus (n=2), pallidum (n=1), parahippocampal gyrus (n=2), pituitary gland (n=1), precentral gyrus (n=1), superior and middle frontal gyri (n=1), parietal cortex (n=1), supramarginal gyrus (n=1), subcallosal cortex (n=1), sagittal stratum and posterior thalamic radiation white matter tracts (n=1), corpus callosum (n=1), and external and internal capsule white matter (n=2).

#### Volume and therapy

**Figure 2:**
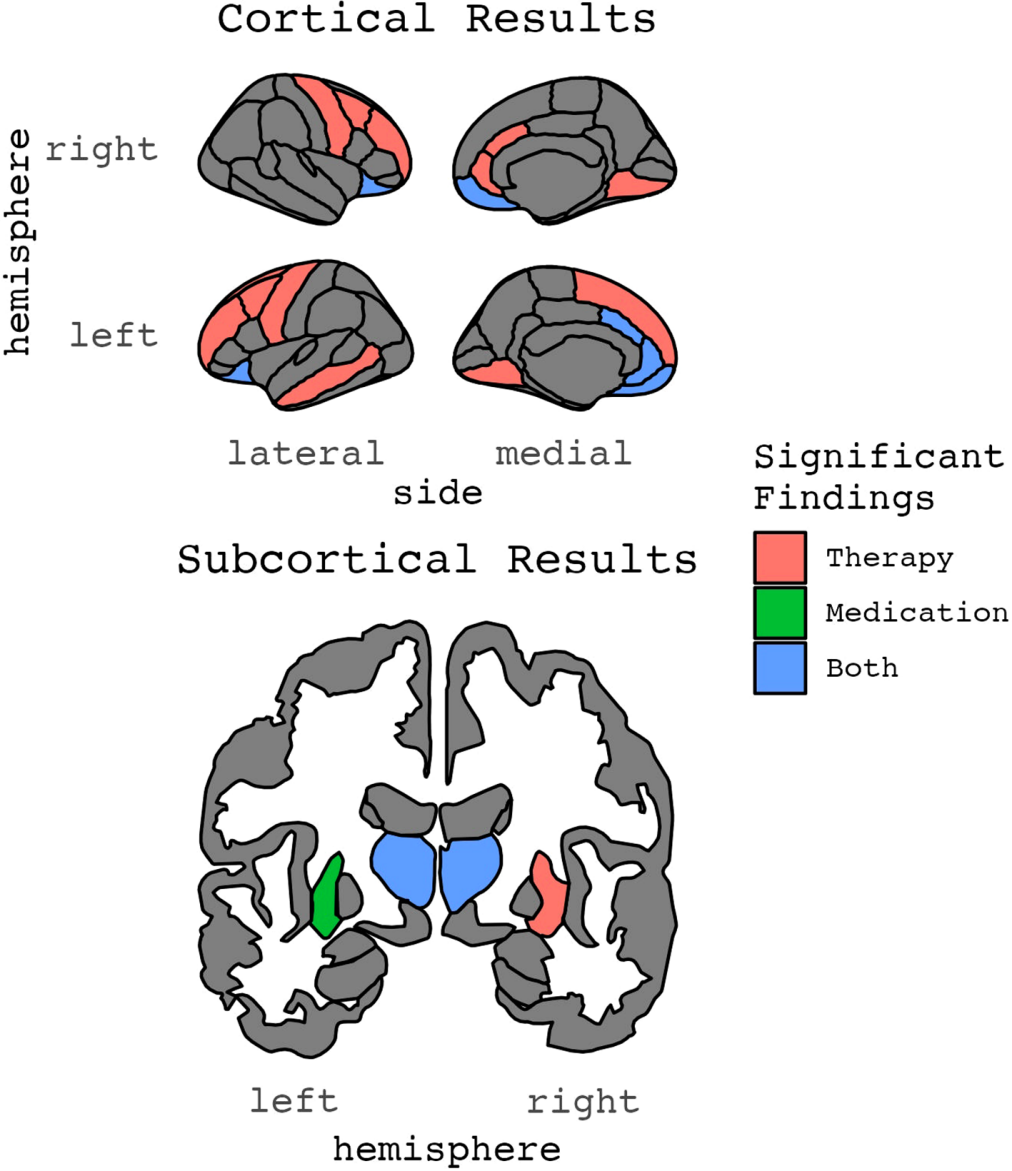
Results for ROIs with two or more significant findings.

**Figure 3:**
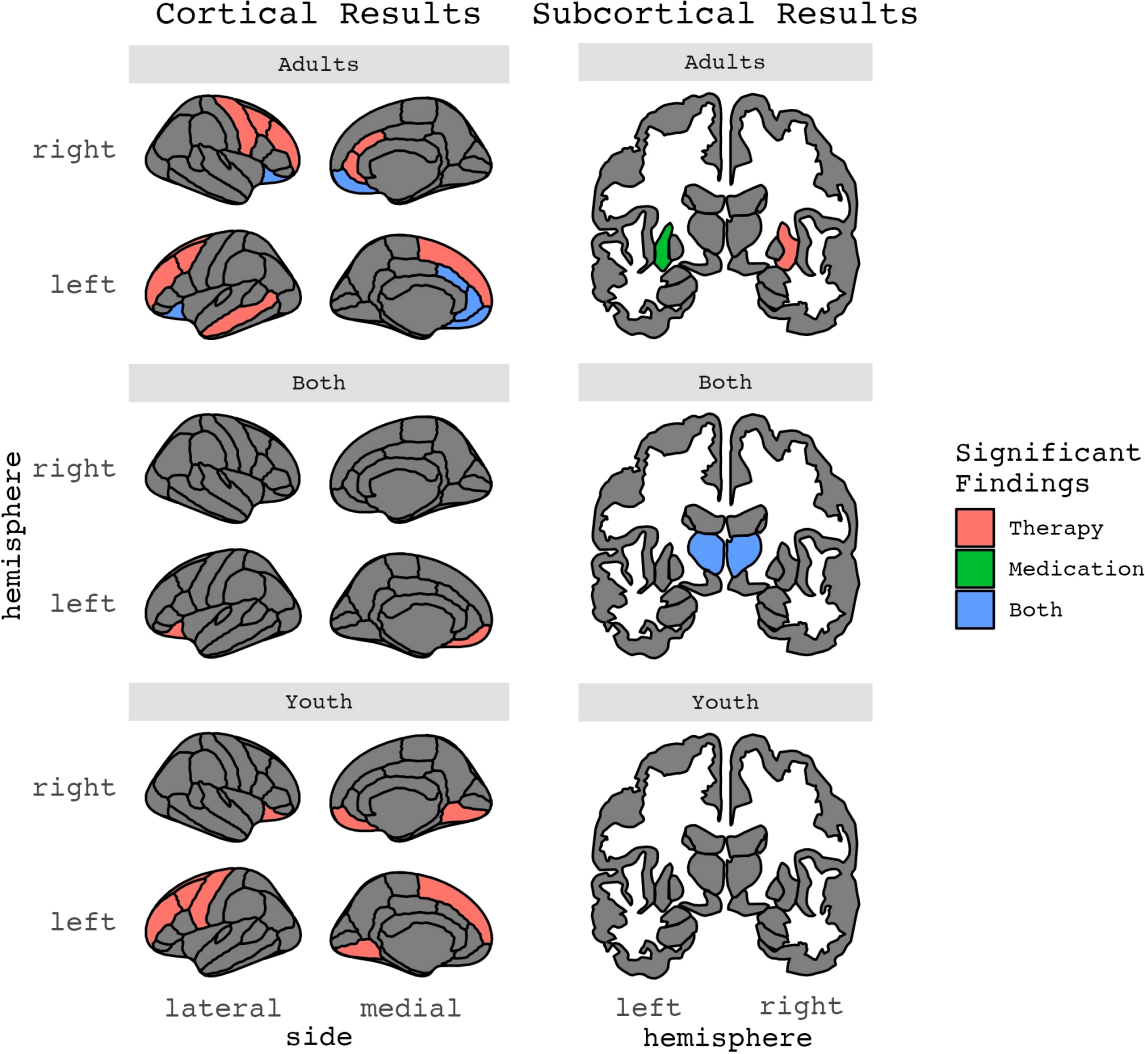
Results for ROIs with two or more significant findings, grouped by age.

**Figure 4:**
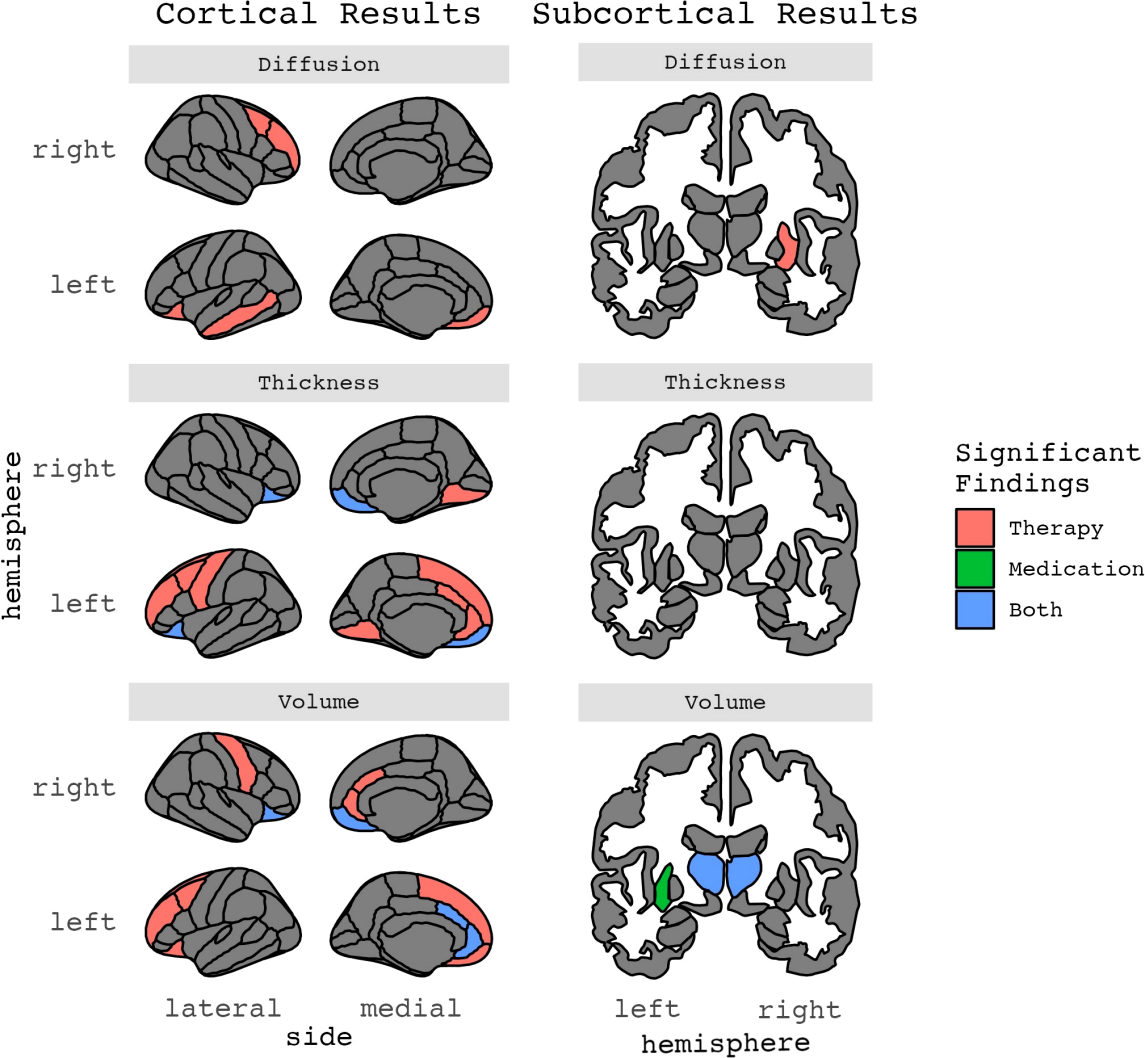
Results for ROIs with two or more significant findings, grouped by structural phenotype.

**Figure 5:**
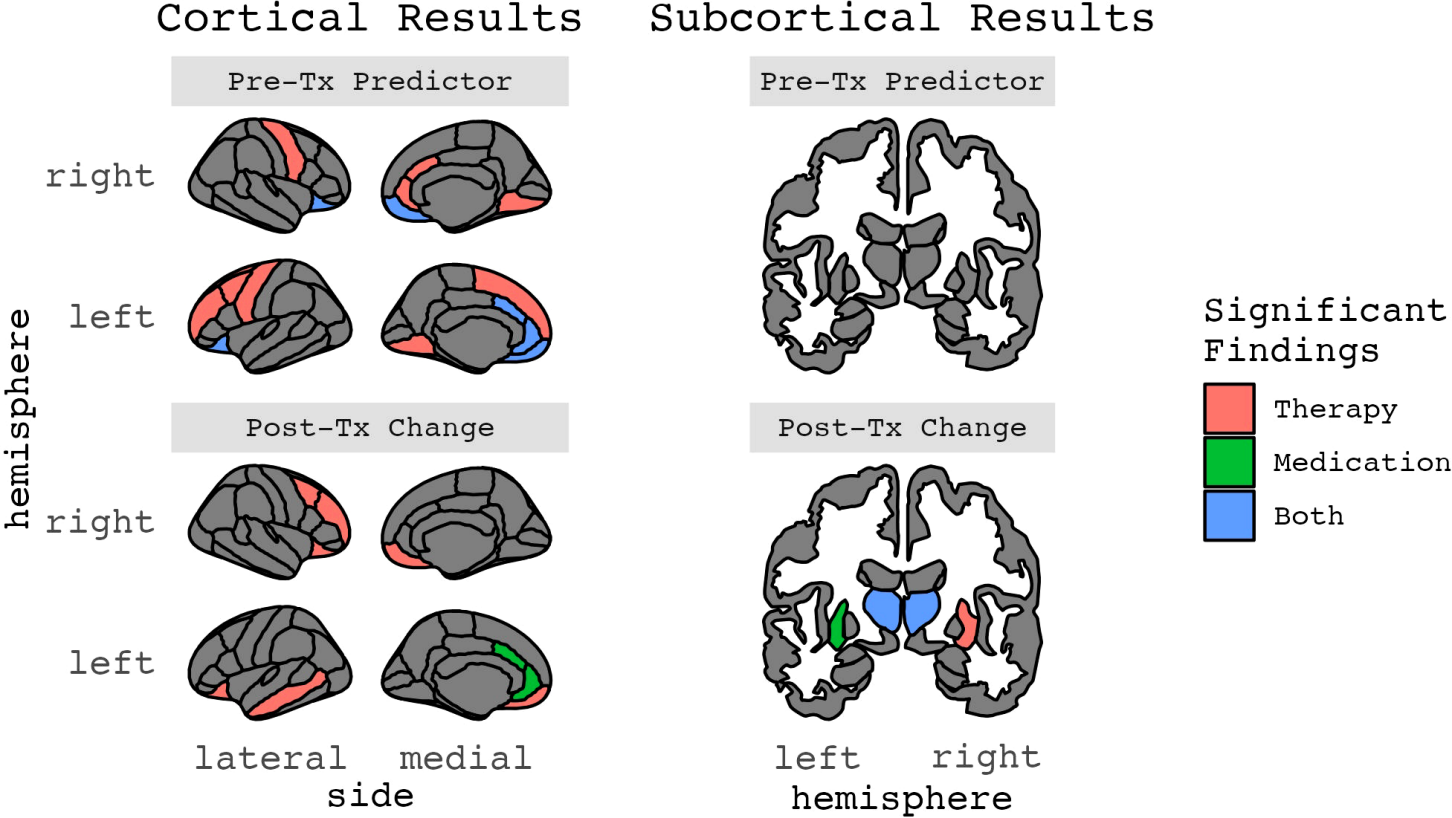
Results for ROIs with two or more significant findings, grouped by whether they were a treatment response predictor or change after treatment (**Figures 2**-**5** in color).

Thirteen publications (n=6 from pediatric samples), from 10 independent samples, estimated whether pre-treatment volume measures were associated with treatment response (n=6; 2 of which were in pediatric samples) or changes in brain volume after therapy (n=7; 4 of which were in pediatric samples) (**Tables 1**–**3**; **Figures 2**–**5**). Total sample sizes ranged from 11 to 160 participants (mean ± SD = 51 ± 38), with OCD group sizes ranging from 11 to 74 before (mean ± SD = 29 ± 17) and after (mean ± SD = 27 ± 17) treatment.

**Table 2:** Study results.

Study	Regions analyzed	Treatment response correlates	Changes after treatment
Psychotherapy studies			
Adult studies			
Brecke et al. (49) *some patients also on medications	Sagittal stratum, posterior thalamic radiation, cingulum bundle. Also did whole-brain analyses *Tract-based Spatial Statistics (TBSS) in FSL. Johns Hopkins University ICBM-DTI-81 white matter atlas.*	None – diffusion measures not associated w/tx response	No sig. changes in diffusion measures
Cao et al. (50)	All *Whole brain tractography. Automated Anatomical Labeling (AAL) atlas, excluding deep white matter voxels and voxels near cerebrospinal fluid*	In tx-responders, decreased nodal clustering coefficient after tx in lh lingual gyrus correlated w/decreased obsessive sx (r=0.52) and in lh fusiform gyrus correlated w/decreased compulsive sx (r=0.62)	Tx-responders’ global clustering coefficient and nodal clustering coefficients of lh lingual, middle temporal, fusiform and precuneus gyri reduced to normal levels. Tx-non-responders had decreased nodal clustering of lh lingual gyrus and lh thalamus and increased nodal shortest path of rh middle occipital gyrus
Zhong et al. (51)	All *Whole brain voxel-based analysis*. *SPM8 and FSL*	Baseline FA values not sig. correlated w/% reduction in Y-BOCS. % change in FA after CBT: in lh middle temporal gyrus, sig. positively correlated w/% reduction in Y-BOCS compulsions subscale (r=0.42); in lh OFC, sig. negatively correlated w/% reduction in Y-BOCS obsessions subscale (r= -0.37); in rh putamen WM, sig. negatively correlated w/% reduction in Y-BOCS compulsions subscale (r= -0.4)	FA values in tx-responders no longer sig. different from controls. Tx responders had increased FA in: lh OFC and middle temporal gyrus, and rh middle frontal gyrus and cerebellum; decreased FA in: rh putamen
Atmaca et al. (52, 53)^†^ *Note: did not correct for multiple comparisons*	Orbitofrontal cortex, thalamus, pituitary gland *Manually traced*	Decreases in Y-BOCS scores sig. positively correlated w/post-tx change in bilateral thalamus vol (Spearman’s ρ: lh=0.65, rh=0.49) and sig. negatively correlated w/post-tx change in lh OFC vol (Spearman’s ρ =-0.47)	Thalamus vol sig. decreased (lh-8%, rh-9%) and lh OFC vol sig. increased (19%) to levels not sig. different from controls. pituitary volume did not change
Tsuchiyagaito et al. (54) *some patients also on medications	All *Whole brain voxel-based morphometry, SPM12*	Larger baseline lh middle frontal vol in tx-responders vs. non-responders	N/A
Hashimoto et al. (55) *all patients also on SSRIs	All *Whole brain voxel-based morphometry, VBM 8 toolbox in SPM8.*	No baseline volumes sig. correlated w/% reduction in Y-BOCS. Tx responder group had larger baseline GM vol in rh vmPFC (subgenual ACC), rh OFC, rh precentral gyrus, lh ACC; WM vol in lh cingulum and superior frontal than non-responder group	N/A
Fullana et al. (56) *all patients also on medications	Medial OFC, rostral anterior cingulate cortex, subcallosal cortex *FreeSurfer v5.1 Destrieux atlas*	No sig. volume predictors of tx response. Thinner baseline lh rACC sig. associated w/greater sx reduction (r=-0.32, explained 8% of variance). Tx responders had sig. thinner baseline lh rACC than non-responders.	N/A
Pediatric studies			
Pagliaccio et al. (57) *4 patients started SSRIs mid-tx	All ROIs *FreeSurfer v6.0 Destrieux atlas.* *MRtrix for structural connectivity (probabilistic tractography).*	No sig. subcortical volume predictors. Thinner cortex in 9 frontoparietal regions (lh angular, middle frontal, superior frontal, precentral and superior temporal gyri; rh anterior insula; bilateral medial occipitotemporal and lingual sulci) and structural connectivity in 10 cingulopercular regions ( lh anterior cingulate cortex, insular cortex, thalamus, putamen, and inferior, middle, and superior frontal sulci) significantly predicted clinical improvement (~ 50% of variance in post-tx CY-BOCS scores above and beyond pre-tx scores.) Left supramarginal gyrus thickness significantly predicted CBT response (Cohen’s d for responders vs. non-responders=1.42; logistic regression: z=-2.22; ROC analysis: 72.2% specificity, 90% sensitivity, AUC=86.7).	N/A
Huyser et al. (58) *some patients on medications after therapy ended	All ROIs, but *a priori* ROIs (OFC GM and external/internal capsule WM) had less stringent multiple testing correction *Whole brain voxel-based morphometry, VBM8 toolbox in SPM8.*	CY-BOCS scores and changes in scores over time not sig. correlated w/volume and changes in volume (respectively) at end of tx or 1.5 years later	Increased OFC GM vol at end of tx and 1.5 years later. When split groups into younger (8-12 y.o.) and older (13-18 y.o.) age groups, only younger one sig.
Huyser et al. (59)	Voxel-wise whole brain and specific ROIs. GM: striatum (caudate, putamen, pallidum), OFC, lateral and medial PFC, ACC, and parietal cortex, supramarginal gyrus; WM: CC, cingulum, capsula interna/externa *Voxel-based morphometry, analyzed w/VBM DARTEL in SPM8. Also identified ROIs of interest using AAL template for GM and ICBM WMPM-152 template for WM*	Post-tx OFC GM vol positively correlated w/decrease in sxs (r=0.50)	OFC GM and external capsule WM vol increased in patients (and decreased in controls), although only sig. w/small vol, not whole-brain, correction
Rosenberg et al. (60)	Thalamus *Manually traced*	Did not test	Thalamus vol did not sig. change
Medication studies			
Adult studies			
Atmaca et al. (61, 62)^†^ *Note: did not correct for multiple comparisons*	Thalamus, OFC, and pituitary gland *Manually traced*	Change in lh thalamus vol sig. correlated w/change in Y-BOCS (r=0.49) but change in pituitary vol was not correlated	Thalamus vol. sig. decreased (avg. 9%, no longer sig. diff. from controls), pituitary gland vol sig. increased (avg. 35%), OFC vol did not change
Tang et al. (63)	All *Whole-brain voxel-based morphometry, SPM8*	Not directly tested, although only rescanned tx responders so reported changes after tx could be tx response correlates	Tx responders had increased vol in lh thalamus, putamen, & anterior and posterior cingulate gyri. Tx non-responders were not rescanned.
Yun et al. (64)	Cortical ROIs *FreeSurfer v5.3 Destrieux atlas*	Support vector machine algorithm successfully differentiated responders and non-responders using pre-tx cortical surface area and thickness ISCs (89% accuracy). Pre-tx thickness ISC between rh dlPFC and lh precuneus critical for classifying responders. Pre-tx surface area ISC between lh anterior insula and intraparietal sulcus critical for classifying non-responders.	N/A
Narayanaswamy et al. (65)	Global GM and OFC, cingulate cortex, dorsolateral PFC, caudate nucleus and globus pallidus *Whole-brain voxel-based morphometry. VBM Toolbox 8 in SPM8. Wake Forest University School of Medicine Pickatlas*	Larger pre-tx lh ACC vol associated w/sig. greater sx improvement	N/A
Fan et al. (66)▽ *Note: did not correct for multiple comparisons, used alpha of 0.001*	LH medial superior frontal gyrus, temporo-parietal lobe, occipital lobe, insula, striatum; RH frontal lobe and midbrain *Voxel-based diffusion analysis in FSL and SPM8.*	Pre-tx lh striatum RD and rh midbrain RD and MD sig. correlated w/decrease in Y-BOCS compulsive scores (rs=-0.71, -0.57, -0.58, respectively) No sig. correlations between baseline or changes in Y-BOCS scores and changes in DTI parameters	Decreased RD of lh striatum (-4%) and rh midbrain (-5%), decreased MD of rh midbrain (-5%)
Yoo et al. (67) *Note: did not correct for multiple comparisons, used alpha of 0.001*	All *Whole-brain voxel-based morphometry, SPM2. Diffusion processing uncertain.*	Changes in FA not sig. correlated w/changes in Y-BOCS	Decreased FA in rh posterior thalamic radiation. While no other sig. changes in FA, post-tx group comparisons showed FA mostly normalized
Pediatric studies			
Szeszko et al. (68)+	Amygdala *Manually traced*	Changes in lh amygdala vol not sig. correlated w/changes in Y-BOCS (was correlated w/dosage and total exposure)	Lh amygdala vol decreased 15% after tx (no longer sig. asymmetry) although still not sig. different from controls.
Gilbert et al. (69)+	Thalamus *Manually traced*	Decrease in thalamus vol after tx sig. correlated w/decrease in sx severity (r=0.74)	Decreased thalamus vol (-19%, effect size = 1.28) to levels comparable to controls
Psychotherapy and medication studies			
Adult studies			
Hoexter et al. (70)	Medial and lateral OFC *FreeSurfer v4.5 Desikan-Killiany atlas*	OFC thickness significantly differentiated tx responders vs. non-responders (logistic regression; accuracy, sensitivity, specificity: ~80%). Thicker lh medial OFC + thinner rh medial OFC associated w/higher probability of being tx-responder	N/A
Hoexter et al. (71)	OFC, ventral and dorsal anterior cingulate, amygdala, hippocampus, parahippocampal gyrus, caudate, putamen, thalamus. Also exploratory whole brain voxel-wise analysis *Voxel-based morphometry in VBM5 Toolbox in SPM5. ROIs identified w/AAL SPM toolbox. Used separate MC correction thresholds for voxel-based results in a priori ROIs and rest of brain*	Larger pre-tx rh medial OFC GM vol (primarily in subgenual ACC) sig. correlated w/% reduction in Y-BOCS after psychotherapy (r=0.81). Smaller pre-tx rh middle lateral OFC GM vol sig. correlated w/% reduction in Y-BOCS after meds (r=0.83)	N/A
Hoexter et al. (72)	OFC, anterior cingulate, temporolimbic cortices (parahippocampal gyrus, amygdala, and hippocampus), striatum, thalamus. Also exploratory whole-brain voxel-wise analysis *Voxel-based morphometry in VBM5 Toolbox in SPM5. ROIs identified w/AAL SPM toolbox. Used separate MC correction thresholds for voxel-based results in a priori ROIs and rest of brain*	None reported	CBT group - no sig. GM vol changes in putamen, mOFC, or ACC. Meds group - sig. increased lh putamen GM vol. Voxel-wise analyses found no sig. changes for either tx.
Pediatric studies			
Vattimo et al. (73)	Caudate nucleus, thalamus, and OFC *FreeSurfer v6.0 Desikan-Killiany atlas*	Trending association for larger pre-tx caudate vol when therapy + meds groups combined (sig. when excluded outlier). Rh caudate vol accounted for 20.2% of variance in post-tx Y-BOCS. No sig. findings when tx groups separated	N/A
Lázaro et al. (74)*	All *Whole-brain voxel-based morphometry, VBM2 toolbox and SPM5.*	Not tested	No sig. changes, although OCD group’s GM vol no longer sig. different from control group.

tx, treatment; sx, symptoms; lh, left hemisphere; rh, right hemisphere; OCD, Obsessive-Compulsive Disorder; OFC, orbitofrontal cortex; FA, fractional anisotropy; RD, radial diffusivity; MD, mean diffusivity; GM, gray matter; WM, white matter; vol, volume; rACC, rostral anterior cingulate; PFC, prefrontal cortex; CC, corpus callosum; Y-BOCS, Yale-Brown Obsessive Compulsive Scale; CBT, cognitive behavioral therapy; ROI, region of interest; MC, multiple comparison; ISC, individualized structural covariance. *All subjects in (74) were given both fluoxetine and exposure and response prevention, so the effects of treatment type cannot be determined. + These samples overlap. Most of the Szeszko et al. (68) sample was part of the Gilbert et al. (69) sample. ▽ For their treatment-related analyses, Fan et al. (66) examined ROIs that were significantly different between their sample’s OCD patients and controls in whole-brain voxel-wise analyses at baseline, so we did not consider these *a priori* regions. † Publications (52, 53) are from the same study and sample. Publications (61, 62) are also from the same study and sample. It is unclear if there is sample overlap between (51/52) and (60/61).

**Table 3:** Brain regions with significant findings in at least two studies.

ROIs	Therapy studies	Findings	Medication studies	Findings
Cortical				
**Orbitofrontal cortex (OFC)** # of publications that examined *a priori* and found significant results: 5/10 # of whole brain publications that found significant results: 2/7	Hoexter 2015^ – bilateral (primarily medial OFC) Zhong – lh (MNI -18,11,-20) Atmaca 2018b^ – lh Huyser 2013^++^^ (lh; medial) + 2014^++^^ (rh; medial) Hashimoto – rh (BA11; medial) VBM Nulls Lázaro^++^ Tsuchiyagaito Hybrid Nulls Hoexter 2012^ (mOFC) Hoexter 2013^ ROI-Based Nulls Vattimo^++^^ Fullana^ (subcallosum) Atlas-Based Nulls Pagliaccio^++^ Cao	Thicker baseline lh + thinner rh associated w/ tx responder Tx-responders had increased FA after tx Vol. increased after tx Vol. increased after tx + 1.5 yrs later Tx-responders had larger baseline vol than non-responders * * *No sig. findings* *No sig. findings* * * *No sig. vol change after tx* *No sig. tx response predictors* *No sig. findings* *Not sig. tx-response predictors* *Not sig. tx-response predictor* *No sig. structural connectivity findings*	Hoexter 2015^ – bilateral (primarily medial OFC) Hoexter 2013^ – rh (ventral portion, BA 10) VBM Nulls Tang Lázaro^++^ Hybrid Nulls Hoexter 2012^ (mOFC) ROI-Based Nulls Vattimo^++^^ Atmaca 2016a^ Narayanaswamy^	Thicker baseline lh + thinner rh associated w/ tx responder Smaller baseline vol associated w/ sx improvement * * *No sig. vol change for tx responders* *No sig. findings* * * *No sig. vol change after tx* * * *No sig. findings* *No sig. vol. change after tx* *No sig. findings*
Anterior cingulate cortex (ACC)* # of publications that examined *a priori* and found significant results: 3/5 # of whole brain publications that found significant results: 2/8	Hashimoto – lh (BA24; caudal or posterior cing.) Hoexter 2013^ – rh (subgenual; BA 25/32) Hashimoto – rh (subgenual; BA25) Fullana^ – lh (rostral) VBM Nulls Zhong Lázaro^++^ Tsuchiyagaito Hybrid Nulls Huyser 2013^++^^ + 2014^++^ Hoexter 2012^ ROI-Based Nulls None Atlas-Based Nulls Pagliaccio^++^ Cao	Tx-responders had larger baseline vol than non-responders Larger baseline vol associated w/ sx improvement Tx-responders had larger baseline vol than non-responders Tx-responders had thinner baseline cortex than non-responders * * *No sig. findings* *No sig. findings* *No sig. findings* * * *No sig. findings* *No sig. vol change after tx* * * * * *No sig. findings* *No sig. findings for tx responders*	Tang – lh (MNI -33,25.5, -9) Narayanaswamy^ – lh (likely caudal anterior; MNI -15, 3, 22) VBM Nulls Lázaro^++^ Hybrid Nulls Hoexter 2012^ Hoexter 2013^ ROI-Based Nulls None	Tx-responders had increased vol after tx Larger baseline vol associated w/ sx improvement * * *No sig. findings* * * *No sig. vol change after tx* *No sig. findings*
**Superior frontal** # of publications that examined *a priori* and found significant results: 0/1 # of whole brain publications that found significant results: 2/11	Pagliaccio^++^ – lh Hashimoto – lh VBM Nulls Zhong Lázaro^++^ Tsuchiyagaito Hybrid Nulls Hoexter 2012 + 2013 Huyser 2013^++^^ + 2014^++^ Atlas-Based Nulls Cao ROI-Based Nulls None	Thinner baseline cortex predicted post-tx improvement Tx-responders had larger baseline WM vol than non-responders *No sig. findings* *No sig. findings* *No sig. findings* * * *No sig. findings* *No sig. findings* *No sig. structural connectivity findings*	VBM Nulls Tang Lázaro^++^ Hybrid Nulls Hoexter 2012 + 2013 Fan^▽^ ROI-Based Nulls None	* * *No sig. vol change for tx responders* *No sig. findings* * * *No sig. findings* *No sig. diffusion changes after tx in adjacent WM*
**Middle frontal** # of publications that examined *a priori* and found significant results: 0/2 # of whole brain publications that found significant results: 3/10	Pagliaccio^++^ – lh Tsuchiyagaito – lh (BA 10, 46) Zhong – rh VBM Nulls Lázaro^++^ Hashimoto Hybrid Nulls Huyser 2013^++^^ + 2014^++^ Hoexter 2012 + 2013 ROI-Based Nulls None Atlas-Based Nulls Cao	Thinner baseline cortex predicted post-tx improvement Tx-responders had larger baseline vol than non-responders Tx-responders had increased FA after tx * * *No sig. findings* *No sig. findings* * * *No sig. findings* *No sig. findings* * * * * * * *No sig. structural connectivity findings*	VBM Nulls Tang Lázaro^++^ Hybrid Nulls Hoexter 2012 + 2013 ROI-Based Nulls Narayanaswamy^ (dlPFC)	* * *No sig. vol change for tx responders* *No sig. findings* * * *No sig. findings* * * *No sig. findings*
**Precentral gyrus** # of publications that examined *a priori* and found significant results: 0/1 # of whole brain publications that found significant results: 2/10	Hashimoto – rh (BA 6) Pagliaccio^++^ – lh VBM Nulls Zhong Lázaro^++^ Tsuchiyagaito Hybrid Nulls Huyser 2013^++^^ + 2014^++^ Hoexter 2012 + 2013 Atlas-Based Nulls Cao ROI-Based Nulls None	Tx-responders had larger baseline vol than non-responders Thinner baseline cortex predicted post-tx improvement *No sig. findings* *No sig. findings* *No sig. findings* * * *No sig. findings* *No sig. findings* * * *No sig. structural connectivity findings*	VBM Nulls Tang Lázaro^++^ Hybrid Nulls Hoexter 2012 + 2013 ROI-Based Nulls None	*No sig. vol change for tx responders* *No sig. findings* * * *No sig. findings*
**Middle temporal** *Not an a priori ROI in any study* # of whole brain publications that found significant results: 2/10	Cao – lh Zhong – lh VBM Nulls Lázaro^++^ Hashimoto Tsuchiyagaito Hybrid Nulls Huyser 2013^++^ + 2014^++^ Hoexter 2012 + 2013 ROI-Based Nulls Pagliaccio^++^ Atlas-Based Nulls None	Tx-responders had decreased nodal clustering coefficient after tx Tx-responders had increased FA after tx * * *No sig. findings* *No sig. findings* *No sig. findings* * * *No sig. findings* *No sig. findings* * * *Not sig. tx response predictor*	VBM Nulls Tang Lázaro^++^ Hybrid Nulls Hoexter 2012 + 2013 Fan ^▽^ ROI-Based Nulls None	*No sig. vol change for tx responders* *No sig. findings* * * *No sig. findings* *No sig. diffusion changes after tx in adjacent WM*
**Lingual gyrus** ***Sulcus specifically*** *** *** *Not an a priori ROI in any study* * * # of whole brain publications that found significant results: 2/11	Cao – lh Pagliaccio^++^ – bilateral VBM Nulls Zhong Lázaro^++^ Hashimoto Tsuchiyagaito Hybrid Nulls Hoexter 2012 + 2013 Huyser 2013^++^ + 2014^++^ ROI-Based Nulls None Atlas-Based Nulls None	Tx-responders had decreased nodal clustering coefficient after tx Thinner baseline cortex predicted post-tx improvement * * *No sig. findings* *No sig. findings* *No sig. findings* *No sig. findings* * * *No sig. findings* *No sig. findings*	VBM Nulls Tang Lázaro^++^ Hybrid Nulls Hoexter 2012 + 2013 ROI-Based Nulls None	* * *No sig. vol change for tx responders* *No sig. findings* * * *No sig. findings*
Subcortical				
Putamen* # of publications that examined *a priori* and found significant results: 1/3 # of whole brain publications that found significant results: 3/8	Zhong – rh VBM Nulls Lázaro^++^ Hashimoto Tsuchiyagaito Hybrid Nulls Hoexter 2012^ Hoexter 2013^ Huyser 2013^++^^ Atlas-Based Nulls Pagliaccio^++^ Cao ROI-Based Nulls None	Tx-responders had decreased FA after tx * * *No sig. findings* *No sig. findings* *No sig. findings* * * *No sig. vol change after tx* *No sig. findings* *No sig. findings* * * *Not sig. tx-response predictor* *No sig. structural connectivity findings*	Tang – lh Hoexter 2012^ – lh Fan – lh (striatum. Likely in putamen but not definite) VBM Nulls Lázaro^++^ Hybrid Nulls Hoexter 2013^ ROI-Based Nulls None	Tx-responders had increased vol after tx Vol increased after tx Radial diffusivity decreased after tx * * * * *No sig. findings* * * *No sig. findings*
Thalamus* # of publications that examined *a priori* and found significant results: 3/7 # of whole brain publications that found significant results: 2/9	Atmaca 2018b^ – bilateral Cao – lh VBM Nulls Zhong Lázaro^++^ Hashimoto Tsuchiyagaito Hybrid Nulls Huyser 2013^++^ + 2014^++^ Hoexter 2012^ + 2013^ ROI-Based Nulls Rosenberg^++^^ Vattimo^++^^ Atlas-Based Nulls Pagliaccio^++^	Vol decreased after tx Tx-non-responders had decreased nodal clustering after tx * * *No sig. findings* *No sig. findings* *No sig. findings* *No sig. findings* * * *No sig. findings* *No sig. findings* * * *No sig. vol change after tx* *No sig. findings* * * *Not sig. tx-response predictor*	Atmaca 2016a^ – bilateral Gilbert^++^^ – lh + rh combined Tang – lh VBM Nulls Lázaro^++^ Hybrid Nulls Hoexter 2012^ + 2013^ ROI-Based Nulls Vattimo^++^^	Vol decreased after tx Vol decreased after tx Tx-responders had increased vol after tx * * *No sig. findings* * * *No sig. findings* * * *No sig. findings*

Gray = additional location information, Orange = volume findings, Green = cortical thickness findings, Blue = diffusion findings, * denotes region in the cortico-striato-thalamo-cortical circuit of OCD, ^++^pediatric study, ^denotes this was an *a priori* ROI in this study. ▽ For their treatment-related analyses, Fan et al. (66) examined ROIs that were significantly different between OCD patients and controls in whole-brain voxel-wise analyses at baseline, so we did not consider these a priori regions. Hybrid nulls refer to null studies that used a combination of voxel-wise and ROI approaches. Brecke et al. (49) and Yoo et al. (67) examined white matter tracts and are not included in this table. Yun et al. (64) is also not included because it reported on structural covariance networks that contain several interdependent regions rather than individual ROIs. Medication studies do not report atlas-based nulls because Yun et al. was the only medication study to use an atlas-based approach. Underlining used to emphasize the direction of effects.

##### Pretreatment brain volume correlates of therapy treatment response

###### Adults

Of four adult studies (n_samples_=4; n_publications_=4), three reported on pre-treatment brain volume correlates of therapy-related symptom change (55, 56, 71) and two reported on pre-treatment differences between therapy responders and non-responders (54, 55). With the exception of Hoexter et al. (71), all of these studies included concurrent medication that originated prior to psychotherapy in at least some or all patients.

Among the three studies of pre-treatment brain structure and symptom change following psychotherapy, two (n_OCD1_ = 74, n_OCD2_ = 39) reported no significant associations between pre-treatment brain volume and therapy-related symptom change (55, 56). The third study of 15 patients treated for OCD, without concurrent medication, found that larger pre-treatment right medial prefrontal cortex gray matter volume (primarily in the subgenual cingulate) was associated with a greater reduction in OCD symptoms following therapy (71).

The two studies that evaluated treatment responders relative to non-responders both identified significant associations. In contrast to null findings described above when analyzing all OCD patients together, Hashimoto and colleagues (2014) found that eventual treatment responders (n=24) were characterized by larger prefrontal gray matter volumes in the bilateral anterior cingulate cortex, right orbitofrontal cortex, and right precentral gyrus in addition to larger white matter volumes in the left cingulum and superior frontal region at baseline when compared to non-responders (n=15) (55). Notably, the observed larger right anterior cingulate gray matter volume aligns with the finding from Hoexter and colleagues (2013) reviewed above. The second study found that responders (n=14) had larger left middle frontal gyrus gray matter volumes at baseline relative to non-responders (n=17) (54).

###### Youth

The two pediatric studies (n_OCD1_ = 28, n_OCD2_ = 29) of volume correlates and predictors of psychotherapy treatment response produced null findings (57, 73). Vattimo and colleagues (73) did note that larger pre-treatment caudate volume was associated with improved treatment response in analyses that combined therapy and medication treatment groups; however, this association was not significant in either the psychotherapy or medication treatment groups when considered independently (73).

##### Therapy-related volume change and treatment response

###### Adults

In adult studies (n_samples_=2; n_publications_=3), Hoexter and colleagues (2012) found no significant changes in gray matter volume associated with therapy (n=13) (72). The other sample (n=12), which examined volumes of the thalamus, orbitofrontal cortex, and pituitary gland only, reported that therapy-related symptom reductions were associated with decreases in bilateral thalamus volume and increases in left orbitofrontal cortex volume (52) but no differences in pituitary gland volume (53).

###### Youth

The four pediatric publications (n_samples_=3) evaluating changes in brain volume in the context of therapy have produced inconsistent findings. One found significant volume increases in the parietal lobes, which normalized to the level of healthy controls, however these patients (n=15) were also on SSRIs during treatment (74). Another study (n=11) found no significant changes in thalamic volume after therapy, the only region of interest (ROI) assessed (60). A third pediatric study of OCD patients found that, much like an adult study (53), treatment was associated with increases in orbitofrontal cortex gray matter volume at the end of therapy (n=26) and one and half years later (n=17) (58, 59). Symptom improvement was positively correlated with orbitofrontal cortex gray matter volume at the completion of CBT but not follow-up (58). Notably, post-hoc analyses that split subjects into younger (8-12 years old) and older (13-19 years old) groups revealed that treatment-related increases in OFC volume were only present among younger patients (58). In the original study reporting on findings immediately following the end of treatment (59), bilateral external capsule white matter volume was also significantly increased in OCD patients (n=26) relative to controls (n=27), however, in the smaller longitudinal sample (n=17 patients, 20 controls)^1^, this was not found at the end of treatment or one and half years later (58).

#### Cortical thickness and therapy

Three studies (n=1 from pediatric samples) examined pre-treatment cortical thickness measures associated with psychotherapeutic treatment response (**Tables 1**–**3**; **Figures 2**–**5**). No identified studies evaluated cortical thickness change in the context of psychotherapy. Total sample sizes ranged from 41 to 160 participants (mean ± SD = 85 ± 65), with OCD group sizes ranging from 28 to 74 before and after treatment (mean ± SD = 44 ± 26).

##### Pretreatment cortical thickness correlates of therapy treatment response

###### Adults

The two identified adult studies reported conflicting results. Fullana and colleagues (56) analyzed cortical thickness in the medial OFC, rostral anterior cingulate cortex, and subcallosal cortex and found that thinner left rostral anterior cingulate cortex at baseline was correlated with greater therapy-related symptom improvement (56). Group comparisons also showed that eventual treatment responders (n=35) had significantly thinner left rACC than eventual treatment non-responders (n=39). It is important to note, though, that these patients were also receiving pharmacotherapy throughout the study. The other adult study (70), unlike Fullana et al. (56), found significant results in the orbitofrontal cortex. In a secondary analysis of a randomized clinical trial comparing fluoxetine and group CBT, Hoexter and colleagues (70) examined the medial and lateral orbitofrontal cortex and found that OFC thickness significantly differentiated treatment responders (n=13, including therapy and medication groups) and non-responders (n=16, including therapy and medication groups), regardless of whether the patient received medication or therapy. Thicker left and thinner right medial OFC were associated with a higher probability of being a treatment responder.

###### Youth

The only pediatric study analyzing baseline cortical thickness measures found that thinner cortex in nine frontoparietal regions, especially the left supramarginal gyrus, significantly predicted clinical improvement after CBT (n=28) (57). The other significant regions included left angular, middle frontal, superior frontal, precentral and superior temporal gyri, along with right anterior insula and bilateral medial occipitotemporal and lingual sulci. These findings remained significant when excluding the four subjects who began taking SSRIs mid-therapy. Left supramarginal gyrus cortical thickness significantly predicted who responded to therapy, with 72.2% specificity and 90% specificity (AUC=86.67), which was significantly better than predictions based on pretreatment CY-BOCS (AUC=63.06). The effect size for the mean difference in supramarginal gyrus thickness between the responder (n=10) and non-responder (n=18) groups was also large (Cohen’s d=1.42).

##### Cortical thickness changes after therapy

No identified studies measured cortical thickness after psychotherapy.

#### Diffusion and therapy

Four studies (n=1 from a pediatric sample) examined whether pre-treatment diffusion metrics, which are measures of white matter properties and structural connectivity, were associated with psychotherapeutic treatment response (n=2) or changes in diffusion metrics after therapy (n=3) (**Tables 1**–**3**; **Figures 2**–**5**). Total sample sizes ranged from 55 to 175 participants (mean ± SD = 94 ± 55), with OCD group sizes ranging from 28 to 85 before treatment (mean ± SD = 45 ± 27) and 26 to 56 after treatment (mean ± SD = 29 ± 17).

##### Diffusion correlates of therapy treatment response

###### Adults

Of the three adult studies (n_samples_=3, n_publications_=3), one found no significant predictors (n_OCD_=26) (49), while the other two (n_OCD1_ = 34, n_OCD2_ = 56) found significant associations between changes in diffusion metrics and decreases in OCD symptoms after CBT but not baseline measures alone and post-treatment symptom scores (50, 51). Still, of note, the percent reduction in the Y-BOCS obsessions and compulsions subscale scores following CBT were significantly correlated with changes in FA and nodal clustering coefficients (**Table 2**).

###### Youth

The only identified pediatric diffusion study (n_OCD_=28) found that lower structural connectivity in ten cinguloopercular regions (anterior cingulate cortex, insular cortex, thalamus, putamen, and inferior, middle, and superior frontal sulci) at baseline predicted 50% of the variance in post-therapy CY-BOCS scores, even when controlling for demographics and pre-treatment severity. However, associations were no longer statistically significant after accounting for comorbid anxiety disorders (57).

##### Diffusion changes after therapy

###### Adults

In adult studies (n_samples_=3, n_publications_=3), Brecke and colleagues’ (49) analyses did not reveal any significant changes in diffusion measures three months after a concentrated ERP treatment (n_OCD post-tx_=26). Zhong and colleagues ((51) reported that CBT responders (n=38) had increased FA after therapy in the left orbitofrontal cortex and middle temporal gyrus and right middle frontal gyrus and cerebellum, as well as decreased FA in the right putamen; these values were no longer significantly different from controls (n=90). Using graph theory techniques, Cao and colleagues (50) reported that after treatment CBT responders (n=26) had significantly decreased global and nodal clustering coefficients in the left lingual, middle temporal, fusiform, and precuneus gyri. Notably, CBT non-responders (n=8) also had significantly decreased nodal clustering coefficients after treatment in the left lingual gyrus, as well as left thalamus, along with increased nodal shortest path length of the right middle occipital gyrus.

###### Youth

No identified pediatric studies measured changes in diffusion after psychotherapy.

### Medication studies

#### Study design summary

##### Studies and samples

The 14 medication OCD treatment outcome publications (n=4 from pediatric samples) originated from ten independent samples (n=3 from pediatric samples) (**Table 1**). One study administered medication and therapy concurrently to all subjects (74). Total sample sizes ranged from 22 to 131 participants (mean ± SD = 44 ± 29), with OCD group sizes ranging from 11 to 56 before treatment (mean ± SD = 25 ± 12) and 10 to 56 after treatment (mean ± SD = 22 ± 13).

##### Treatments

Samples contained patients who were: drug naïve (n_samples_=6, n_publications_=10); previously treated with medication but not currently using medication or never having used medication to treat OCD (n_samples_= 3, n_publications_=3); or not taking medication within the past four weeks but the status of previous usage was not described (n_samples_=1, n_publications_=1). Regarding previous or current psychotherapy, five publications (n_samples_=3) explicitly stated that OCD patients had not previously received any therapy (n_samples_=2, n_publications_=2) or more than 12 sessions of CBT (n_samples_=1, n_publications_=3). Three studies (n_samples_=3) also reported that patients did not receive therapy while taking medications and a fourth stated that concurrent supportive or family therapy, but not CBT, was allowed.

##### Brain phenotypes and analytic strategy

Four studies (n_samples_=4) estimated associations across the whole brain using whole-brain voxel-based morphometry (n_publications_=2) or voxel-based diffusion analyses (n_publications_=1) with another analyzing all atlas cortical ROIs. Six publications (n_samples_=3/4: Szeszko et al. (68) and Gilbert et al. (69) were largely, but not completely, overlapping samples) analyzed specific ROIs only. Four publications (n_samples_=3) used both ROI and whole brain voxel-wise analyses. The following specific ROIs were examined across studies (n=number of publications): orbitofrontal cortex (n=6), thalamus (n=5), caudate (n=4), amygdala (n=3), anterior cingulate cortex (n=3), putamen (n=2), hippocampus (n=2), parahippocampal gyrus (n=2), pallidum (n=1), pituitary gland (n=1), and dorsolateral prefrontal cortex (n=1).

#### Volume and medication

Ten publications (n=4 from pediatric samples) from seven independent studies estimated pre-treatment volume measures associated with pharmacological treatment response (n_publications_=3; 1 of which was in a pediatric sample) or changes in brain volume after pharmacotherapy (n_publications_=7; 3 of which were in pediatric samples) (**Tables 1**–**3**; **Figures 2**–**5**). Total sample sizes ranged from 22 to 74 participants (mean ± SD = 37 ± 16), with OCD group sizes ranging from 11 to 38 before treatment (mean ± SD = 22 ± 9) and 10 to 29 after treatment (mean ± SD = 19 ± 8).

##### Pretreatment brain volume correlates of medication treatment response

###### Adults

In adult studies (n_samples_=2; n_publications_=2), larger baseline left anterior cingulate cortex volume was correlated with greater symptom reduction after two to five years of SSRI treatment (n=29) (65). A therapy study found similar findings, though they were in the right hemisphere (55). The second study reported that smaller baseline right middle lateral orbitofrontal cortex volume was associated with greater symptom reduction after twelve weeks of fluoxetine (a type of SSRI) treatment (n=14) (71).

###### Youth

The only identified pediatric study found a trending association for larger pre-treatment caudate volume in analyses that combined therapy and medication treatment groups (n=29), with the right caudate accounting for 20.2% of the variance in Y-BOCS score changes after treatment (73). However, this finding did not hold in follow-up analyses testing the two treatment groups separately (n_medication_=12, n_therapy_=17).

##### Volume changes after medication

###### Adults

Of the adult studies (n_samples_=3; n_publications_=4), one found decreased thalamic volume after pharmacotherapy (n=14) (61), while another study found increased volume after treatment (63). However, Tang and colleagues (63) only rescanned patients who were deemed treatment responders (defined as greater than 50% decrease in Y-BOCS score, n=11), which could be driving this discrepancy. Atmaca and colleagues (61) reported that the changes in left thalamic volume were significantly correlated with changes in symptom severity after 12 weeks of SSRI or clomipramine treatment. Other identified volumetric changes after medication included larger pituitary gland (62), left putamen (63, 72) and left anterior and posterior cingulate gyri (63), although, again, the findings from Tang and colleagues were restricted to scans from treatment responders.

###### Youth

Of the pediatric studies (n_samples_=2; n_publications_=3), one found no statistically significant changes in volume after treatment, although the patients (n=15) received both therapy and medication, so the effect of medication cannot be ascertained (74). Two others, based on substantially overlapping samples (n_1_ = 10, n_2_ = 11) but examining two different ROIs, found decreased thalamic (69) and left amygdala (68) volumes after 16 weeks of paroxetine treatment, decreasing by 19% and 15% respectively. Gilbert and colleagues (69) found that the observed decreases in thalamic volume were correlated with greater symptom improvement, while Szeszko and colleagues (68) did not find a significant association between the observed decreases in left amygdala volume and changes in symptom levels.

#### Cortical thickness, surface area, and medication

Two identified studies, both from adult samples, assessed whether pre-treatment cortical thickness measures were associated with treatment response to medications (**Tables 1**–**3**; **Figures 2**–**5**). One of these studies also examined surface area metrics. No identified studies evaluated changes in cortical thickness or surface area after medication treatment. Total sample sizes ranged from 41 to 131 participants (mean ± SD = 86 ± 64), with OCD group sizes ranging from 29 to 56 before and after treatment (mean ± SD = 43 ± 19).

##### Pretreatment cortical thickness and surface area correlates of medication treatment response

In one study, a support vector machine classified OCD patients (n=56) receiving pharmacotherapy as responders (i.e., > 35% decrease in Y-BOCS) or non-responders with 89% accuracy based on combinations of pre-treatment cortical thickness and surface area individualized structural covariances (ISCs) included as feature sets in the classifier (64). This classifier included five cortical surface area-based and seven cortical thickness-based ISCs. Yun and colleagues found that the cortical thickness ISC between the right dorsolateral prefrontal cortex and left precuneus was critical for successfully classifying responders, while the cortical surface area ISC between left anterior insula and intraparietal sulcus was critical for successfully classifying non-responders.

The second study, a secondary analysis of an RCT comparing fluoxetine and group CBT treatments for OCD, described in the therapy section above, analyzed the medial and lateral orbitofrontal cortex and found in a logistic regression model that baseline OFC thickness significantly differentiated treatment responders (n=13, including therapy and medication groups) and non-responders (n=16, including therapy and medication groups) (accuracy 79.3%, sensitivity 77%, specificity 81%, area under the receiver operating curve of 0.88), regardless of whether the patient received medication or therapy (70). Thicker left and thinner right medial OFC were associated with a higher probability of being a treatment responder.

##### Cortical thickness and surface area changes after medication

No identified studies measured changes in cortical thickness or surface area after medication treatment.

#### Diffusion and medication

Two identified studies, both adult samples, estimated pre-treatment diffusion measures associated with treatment response (n=1) or changes in diffusion metrics after pharmacotherapy (n=2) (**Tables 1**–**3**; **Figures 2**–**5**). Total sample sizes ranged from 26 to 50 participants (mean ± SD = 38 ± 17), with OCD group sizes ranging from 13 to 27 before treatment (mean ± SD = 20 ± 10) and 13 to 15 after treatment (mean ± SD = 14 ± 1).

##### Pretreatment diffusion correlates of medication treatment response

Fan and colleagues (66) reported that baseline RD values in the left striatum and right midbrain, as well as baseline MD values in the right midbrain, were significantly correlated with decreases in Y-BOCS compulsive subscale scores after 12 weeks of SSRI treatment (n=15). It is unclear if Yoo et al. (67) (n=13) examined whether baseline diffusion measures were associated with treatment response, however they reported that changes in FA after 12 weeks of citalopram treatment did not correlate with changes in Y-BOCS scores.

##### Diffusion changes after medication

One study (n=15) reported no significant changes in FA values after 12 weeks of SSRI treatment (66) while the other (n=13) reported that right posterior thalamic radiation FA values decreased after 12 weeks of citalopram treatment (67). For Yoo and colleague’s patients, higher FA values, compared to controls, observed at baseline were mostly normalized after treatment, except in the left corpus callosum and right superior temporal region. Right midbrain RD and MD, along with left striatum RD, which had all been significantly higher in patients than controls at baseline, also decreased significantly after pharmacotherapy (66).

## Discussion

Our systematic review of MRI-derived brain structure correlates of psychotherapeutic and pharmacologic treatment response among patients with OCD revealed that despite some intriguing correlations, there is limited consistency across studies. Regions of the cortico-striatal-thalamocortical circuit, including the orbitofrontal and anterior cingulate cortices, thalamus, and putamen, predicted treatment response at baseline or changed longitudinally alongside treatment. However, caution is needed in drawing strong conclusions from the existing literature as sample sizes were mostly small (i.e., OCD groups ranging from 11 to 74) and replication was rare.

Indeed, no regions showed replication in the same hemisphere and imaging and treatment modalities across more than two studies. The lack of consistency in this literature may reflect the limited association between treatment response and brain structure and/or methodological differences including heterogeneity in analyses (e.g., divergent regions of interest examined), patients (e.g., pediatric, adult), and treatment regimens as well as low samples that would not permit the reliable estimation of associations that are small in magnitude. Below, we first describe overall trends in study findings as well as tentative evidence linking variability in cortico-striatal-thalamocortical structure to treatment response and potential mechanisms to explain these findings. We then discuss potential factors that may contribute to the limited number of consistent findings and highlight opportunities for this field including collaboration to boost sample sizes combined with novel multivariate analyses. While neuroimaging studies of treatment response have the potential to identify individual difference factors that may help personalize medicine and identify mechanisms of therapeutic response that may be leveraged to improve future treatments, larger samples will be needed before results will be sufficiently reliable.

### Emerging patterns

#### Overall trends

The majority of studies found that some form of regional brain volume was associated with treatment response. Overall, numerous brain regions showed larger baseline volume associated with better treatment response [right OFC (55), bilateral ACC (55, 65, 71), left middle frontal gyrus (54), right precentral gyrus (55), left cingulum (55), left superior frontal white matter (55)]. Only one region, right orbitofrontal cortex, exhibited smaller baseline volume associated with better treatment response (71), although Hashimoto and colleagues (55) found the opposite. In contrast to volume, where larger was generally better, three studies examining cortical thickness all found thinner cortex at baseline was associated with improvement after treatment [left rostral ACC (56), right OFC (70), left angular, middle frontal, superior frontal, precentral and superior temporal gyri, right anterior insula, bilateral medial occipitotemporal and lingual sulci (57)] (although one study (70) also reported thicker cortex for the left OFC). On average, half or fewer of publications looking a priori at a region of interest reported significant results for that brain region.

#### Anterior cingulate, orbitofrontal cortex, thalamus, and putamen

While studies reported varying and sometimes conflicting results, key structures of the cortico-striato-thalamo-cortical circuit were implicated in many findings – the anterior cingulate, orbitofrontal cortex, thalamus, and putamen. The anterior cingulate is likely involved in action monitoring, error detection, and the expression of fear responses, which have all been implicated in OCD (20). Three out of five publications examining the ACC a priori reported significant results. Larger baseline anterior cingulate volume was correlated with better treatment response for both psychotherapy (55, 71) and pharmacotherapy (65) in three separate studies and in another treatment responders had increased ACC volume after treatment (63). Interestingly, however, thinner left ACC was found to be correlated with better response to psychotherapy (56). Meanwhile, the orbitofrontal cortex has been connected with reward processing and motor and response inhibition, a key component of compulsions (20). Five out of ten publications examining the OFC a priori reported significant results. Two studies found that OFC volume increased after therapy in both children (58, 59) and adults (53). Several studies also reported that OFC structural measures were correlated with treatment response, although the imaging phenotype, laterality, and direction of effects were inconsistent. In one study, larger right OFC baseline volume predicted better response to therapy (55), while in another decreases in left OFC volume after therapy were correlated with greater improvement (52). Further, smaller right OFC baseline volume was associated with better response to medications (71), the opposite direction of the therapy finding. Meanwhile, a cortical thickness study found that thicker left but thinner right medial OFC at baseline predicted better treatment response, regardless of modality (i.e., therapy or medication) (70). OFC white matter may also be affected by therapy, as Zhong and colleagues (2019) (51) found increased fractional anisotropy of the left OFC after therapy.

The thalamus relays sensory information as well as information between cortical and subcortical brain structures and possibly aids cognitive integration (75). Three out of seven publications examining the thalamus a priori reported significant results. Three studies reported decreased bilateral thalamic volume after treatment, including therapy (52) and medication (62, 69). However, another study, that only rescanned treatment responders (n=11), reported increased left thalamus volume after pharmacotherapy (63). Finally, the putamen is involved in motor function and other aspects of goal-directed behavior (76). One out of three publications examining the putamen a priori reported significant results. Left putamen volume increased after SSRI treatment in two studies (63, 72).

#### Potential neural mechanisms

The mechanisms through which treatment may be related to brain structure are still up for debate. Treatment may normalize structural abnormalities or create compensatory neural mechanisms to address the abnormalities. Evidence suggests that SSRIs, the primary class of medications for OCD, change synaptic transmission, post-synaptic transcription growth factors and stimulate neurogenesis (35–38). Meanwhile, psychotherapy, especially cognitive-behavioral therapy and its exposure and response prevention (ERP) treatment for OCD, involves learning new cognitive and behavioral strategies, and learning processes have been linked to structural changes in the brain (39). In addition, fear extinction recall abilities, a key component to ERP success, have been associated with brain structure (specifically ventromedial prefrontal cortex thickness), so it is plausible that improvements in extinction recall following ERP would be correlated with changes in identified brain structures (40). Associations may also reflect neural changes following symptom reduction rather than a mechanism through which treatment causes change.

It is important to note that confounding factors besides treatment could also be influencing measured structural differences. Hydration levels, time of day, and head motion may affect structural MRIs (77–80). Successful treatment may also lead to improved health behaviors, such as better nutrition, hygiene, and exercise, which could themselves be linked to changes in brain structure (81, 82). Additionally, brain structure naturally changes across development (83) and most studies included subjects spanning wide age ranges (e.g., 18-65), which introduces age as a confound.

### Potential drivers of limited associations

#### Small effects and underpowered studies?

MRI-derived indices of brain structure are typically associated with behavior at small levels of effect that require large samples (e.g., Ns>8,000) to detect (84, 85). Such sample sizes are infeasible for individual treatment outcome studies and will require collaborative consortia to detect, such as ENIGMA and the Psychiatric Genomics Consortium (PGC). The small sample sizes of the current literature make it very likely that studies were underpowered, resulting in a host of issues such as imprecise association estimates, imprecise estimated effect sizes, low reproducibility, and reduced chances of detecting a true effect or, conversely, that “detected” effects are indeed true. Unfortunately, small sample sizes have historically been a common-place problem in neuroimaging studies, not just the ones included in the current review (86). One piece of evidence that suggests the current studies are underpowered is the discrepancy in findings for pretreatment patient versus control analyses for these small studies (not described in the current paper) compared to the large ENIGMA consortia results (described above in the Introduction). Additionally, there was within-sample inconsistency wherein removing some participants [e.g., for longitudinal analyses) resulted in differing findings (e.g., (58)]. The small samples and lower power, along with overfitting, may also explain why some effect sizes were extremely large (e.g., r >0.5). Guidelines for interpreting effect sizes vary; nevertheless, one set of recommendations based on 125 meta-analyses in psychology and psychiatry suggest that the Pearson correlation for most clinically important variables falls between 0.15 and 0.3 (87). For neuroimaging phenotypes, data from one of the largest neuroimaging and health studies, the UK Biobank, suggest that variance explained (i.e., R^2^) is commonly around 1% (88).

Even with sufficiently powered samples, it remains possible that MRI may have inadequate spatial resolution to detect changes associated with treatment. Given that the synaptic clef is approximately 20-40 nanometers wide, changes at the synaptic level, the primary mechanism of psychopharmacology, might be beyond the spatial resolution of MRI. Structural changes may be occurring on the nano- and micrometer, not millimeter, scale. Few studies in the existing literature acquired newly possible high-resolution data (i.e., higher than 1 millimeter cubed), and approximately two-thirds analyzed data from lower field strength (i.e., 1.5T) scanners, so these studies may especially be limited in their ability to detect treatment-related changes. Thus, translational models in non-human animals may be useful to test such putative mechanisms for pharmacological treatment [e.g., (89, 90)].

#### Methodological differences

The wide array of methodologies employed in the reviewed studies reduces the pool of studies available to replicate findings. Beyond which brain structure metric was chosen, whether it be volume, cortical thickness, surface area, or diffusion-based measures, there are also different conceptual approaches, particularly voxel-based morphometry versus region-of-interest techniques, numerous software packages that implement these approaches and techniques in varying ways, and the numerous other “researcher degrees of freedom” (91) involved in a study. Many studies only looked at one or a few a priori ROIs, which often did not overlap between studies, making it difficult to determine whether identified findings would be replicated in other studies.

#### Confounding factors

##### Comorbidities

Studies varied on whether they included OCD patients with comorbid psychiatric disorders, and if so, which ones. 20 of the 26 publications analyzed samples that included comorbidities. Some permitted any coexisting disorder besides psychosis, while others allowed only comorbid depression. The remainder varied in which comorbidities were allowed. While OCD patients often meet criteria for other psychiatric disorders as well [e.g. 73-92% lifetime comorbidity rate (4, 92, 93)], increasing the external validity of such studies, other disorders will also exert effects on treatment efficacy, and possibly brain structure, making it difficult to isolate which findings can be attributed to OCD specifically. For example, a study comparing OCD patients with and without Major Depressive Disorder (MDD) found that patients with comorbid MDD had larger reductions of medial OFC gray matter volume than those with OCD alone (94). The ENIGMA OCD studies reported that adult OCD patients with comorbid anxiety had larger intracranial volume than those without comorbid anxiety but no group differences in cortical thickness or surface area for comorbid anxiety or depression (26, 27). However, they caution that these findings came from small samples, as many ENIGMA samples did not assess for or excluded patients with comorbidities, and separate ENIGMA studies of these disorders have reported associated structural abnormalities [e.g. (95)].

##### Other treatments

Some therapy studies (n=6) allowed OCD patients to concurrently be on psychoactive medications, although most required the dosage to remain stable throughout therapy. Indeed, one of these studies provided both therapy and medications to treatment-naïve pediatric patients (74). Multiple treatments confound findings and may have obscured patterns that otherwise would have proven consistent if all studies were restricted to a single treatment. It also makes it difficult to detect if effects differ for therapy versus medication. Two studies (n=4 publications) conducted randomized trials to compare therapy and medications (70–73). However, treatment group sizes were modest (n=12-19), limiting the ability to draw conclusions from these comparisons.

Studies also varied as to whether participants were treatment naïve, so baseline scans could be affected by previous treatment. Results from the ENIGMA studies suggest that medication status can impact structural findings as all but one observed difference between OCD patients and controls was no longer significant when comparing unmedicated patients and controls (26–28).

### Moving forward with large samples and multivariate analyses

Larger samples are needed to understand brain structural correlates of OCD treatment; such samples may be achieved through consortia science wherein many smaller studies are pooled [e.g., ENIGMA, PGC (24, 96)]. In addition to being adequately powered to detect the expected small effects, consortia efforts can reduce methodological differences and other confounding factors such as comorbidities and polytreatment by providing sufficiently large subgroups for analyses that are not impacted by these factors.

Multivariate approaches that consider brain networks or all brain regions simultaneously can also better account for the dependent nature of brain structure, improving our ability to detect small effects (97). A recent review found that neuroimaging studies using multivariate pattern analysis techniques to classify OCD diagnosis had accuracies between 66-100% (98). Many of the treatment “prediction” studies in the current review analyzed data at a group level. For biomarkers to be used clinically, individual-level predictions will be needed using approaches such as logistic regression or machine learning. Machine learning techniques can adeptly implement multivariate analyses and provide data-driven predictions. However, this will require larger samples to produce generalizable results, which reinforces the need for future research using larger samples.

### Strengths and limitations of this systematic review

The large amount of between-study heterogeneity in design and analytic approach prevented us from conducting a meta-analysis. It also weakens our ability to draw strong conclusions from the literature. Another limitation of the current review is that unpublished or non-English studies were excluded, potentially leaving out relevant findings. While the “file drawer” problem is certainly problematic, the peer review process of publication theoretically should increase the level of rigor for included studies. In addition, the authors did not have the resources to adequately translate any potential non-English search results to determine if such studies otherwise met inclusion criteria.

Despite these limitations, the current systematic review holds many strengths as well. First, to our knowledge, this is the most comprehensive and up-to-date review of structural neuroimaging markers of treatment for OCD. Most existing reviews restricted their scope to either psychotherapy or pharmacotherapy, but not both. In addition, few included structural studies, with the majority only reporting on one or two structural studies at most. Second, we utilized reproducible and transparent systematic review processes, guided by PRISMA recommendations (45), to minimize the reporting biases that can exist in narrative literature reviews. Third, all studies used the same treatment outcome measure, the Y-BOCS, allowing for direct comparisons of treatment response across studies. Few other psychiatric disorders have a literature that utilizes a singular assessment tool, making it harder to conduct such direct comparisons. The OCD literature is very fortunate in this regard.

## Conclusions

Our systematic review of brain structure correlates of psychotherapeutic and pharmacologic treatment response among patients with OCD revealed little consistent evidence that brain structure is associated with treatment response. Although structural neuroimaging markers are not yet clinically useful for OCD treatment prognosis or planning, further research is warranted. While potentially small effects and expense could place MRI behind other putative treatment response indicators (e.g., self-report, clinical assessments, genomics), MRI can provide unique mechanistic insights if successful treatment is associated with changes in the brain. In addition, the availability of structural brain scans in medical records and automated procedures being developed to evaluate brain structure in standard radiology workflows heighten the potential for more immediate clinical utility of structural MRI.

Future research should focus on consortia-based larger samples, higher resolution MRI data, and multivariate approaches to confirm our findings and identify other consistent and well-powered findings. There is also a need for more studies assessing cortical thickness, surface area, and diffusion, as these provide information on neurobiological processes distinct from volume which may be differentially altered by treatment for OCD. Finally, greater consistency in ROIs examined in future studies would allow for a meta-analysis to add quantitative insights to the debate on using structural neuroimaging markers in a personalized medicine approach to OCD treatment.

## Footnotes

1 Patients lost to follow-up did not differ from remaining patients in terms of age, gender, symptom severity or change in symptom severity after CBT.
