# Supplementary material for: A systematic review of structural neuroimaging markers of psychotherapeutic and pharmacological treatment for obsessive-compulsive disorder
Source: Front Psychiatry. 2025 Feb 13;15:1432253. doi: 10.3389/fpsyt.2024.1432253 (PMC11865061; doi:10.3389/fpsyt.2024.1432253)
Supplement: Supplementary file 1 [file DataSheet1.docx]

| **Population** | **Intervention** | **Comparison** | **Outcome** | **Study Design** |
| --- | --- | --- | --- | --- |
| Obsessive Compulsive Disorder (OCD) | Treatment/Treatment response/Treatment outcome  Therapy/psychotherapy/counseling  Intervention  Empirically supported treatment (EST)/Empirically based treatment (EBT)  Medication/psychopharmacology/pharmacotherapy  Cognitive behavioral therapy  Exposure and response prevention/Exposure therapy  SSRIs  Brain stimulation/Deep brain stimulation (DBS)/transcranial magnetic stimulation (TMS)/electroconvulsive shock therapy (ECT)/vagus nerve stimulation  Psychosurgery/cingulotomy/capsulotomy | Treatment and control/placebo groups | Neuroimaging  Magnetic resonance imaging/MRI  Structural magnetic resonance imaging (structural MRI)  Volume/Surface area/cortical thickness  Diffusion/Diffusion Tensor Imaging (DTI)/Diffusion Weighted Imaging (DWI) | Data collected before treatment begins |

OR

"obsessive compulsive disorder" OR “obsessive-compulsive disorder”

**AND**

Treatment OR “treatment response” OR “treatment outcome” OR therapy OR “psychotherapy” OR counseling OR intervention OR “empirically supported treatment” OR “empirically based treatment” OR medication OR psychopharmacology OR pharmacotherapy OR “cognitive behavioral therapy” OR “exposure and response prevention” OR “exposure therapy” OR “SSRIs” OR “neurostimulation” OR “deep brain stimulation” OR “DBS” OR “transcranial magnetic stimulation” OR “TMS” OR “electroconvulsive shock therapy” or “ECT” OR “vagus nerve stimulation” OR “psychosurgery” OR “capsulotomy” OR “cingulotomy”

**AND**

Neuroimaging OR “magnetic resonance imaging” OR “MRI” OR “structural magnetic resonance imaging” OR “structural MRI” OR “volume” OR “surface area” OR “cortical thickness” OR diffusion OR “diffusion weighted imaging” OR “diffusion tensor imaging”

**NOT**

“osteochondritis dissecans”

(NOTE – In PsycInfo, the hash mark (#) is used to indicate there might be an additional letter in alternate spellings (e.g. in British spelling)

AND
