# Supplementary material for: A systematic review of structural neuroimaging markers of psychotherapeutic and pharmacological treatment for obsessive-compulsive disorder
Source: Front Psychiatry. 2025 Feb 13;15:1432253. doi: 10.3389/fpsyt.2024.1432253 (PMC11865061; doi:10.3389/fpsyt.2024.1432253)
Supplement: Supplementary file 2 [file DataSheet2.docx]

**Abstract Screening Tool**

For all questions below, answer “yes”, “no”, or “maybe/unsure”. Any question answered “no” is an immediate drop. Do not answer any further questions after the first “no”.

1. **Primary research**: Is the abstract *not* a review of research (i.e., literature review, systematic review, meta-analysis)?
2. **Study design:** Does the study include multiple subjects (i.e. not a case study)?
3. **Language**: Is the abstract written in English?
4. **Population**: Does the study investigate Obsessive Compulsive Disorder (OCD)?
5. **Treatment Study**: Is the effects of one of the following interventions studied?
   1. psychotherapy
   2. psychiatric medications
   3. brain stimulation treatment (ECT, DBS, TMS, etc.)
   4. psychosurgery (e.g. cingulotomy)
6. **Brain Structure Study**: Is the study looking at brain structure? These terms indicate brain structure was investigated:
   1. Morphometry or voxel-based morphometry (VBM) or morphometrics
   2. Volume or gray matter volume
   3. Surface area
   4. Cortical thickness
   5. Structural connectivity or diffusion (DWI, DTI) or tractography or white matter integrity or fractional anisotropy (FA) or radial diffusivity (RD) or mean diffusivity (MD)

Decision: **Keep** (all “yes” or “maybe/unsure” answers) or **Drop** (at least one “no” answer)
